# Air bag-embedded MIL-101(Fe) metal-organic frameworks for an amplified tumor microenvironment activation loop through strategic delivery of iron ions and lentinan

**DOI:** 10.7150/thno.99303

**Published:** 2024-09-09

**Authors:** Tao Han, Yan Sun, Xi Jiang, Chengming Gong, Fei Kong, Yi Luo, Chang Ge, Congyan Liu, Yuping Liu, Yanfei Mou, Huangqin Zhang, Jianming Ju, Yan Chen, Ding Qu

**Affiliations:** 1 Affiliated Hospital of Integrated Traditional Chinese and Western Medicine, Nanjing University of Chinese Medicine, Nanjing, 210028, China.; 2 Jiangsu Province Academy of Traditional Chinese Medicine, Nanjing, 210028, China.; 3 Jiangsu Cancer Hospital, Nanjing, 210002, China.

**Keywords:** Metal-organic frameworks, Triple-negative breast cancer, Tumor microenvironment, Polysaccharides, Tumor-associated macrophages

## Abstract

**Background:** Iron-based nanocarriers have demonstrated potential in redirecting tumor associated macrophages (TAMs) polarization towards the M1 phenotype, critical for activating the tumor microenvironment (TME) in triple negative breast cancer (TNBC). However, their real-world effectiveness is curtailed by insufficient Fe^2+/3+^ exposure and the absence of suitable synergists in tumors.

**Methods:** We introduce an air bag-embedded iron-based MIL-101 metal-organic frameworks (MOF_MIL-101(Fe)_) for igniting the TME in TNBC through bubble-driven tumoral codelivery of Fe^2+/3+^ and lentinan. This system, named HM/Ef/LNT-MOF_MIL-101(Fe)_, features nano-sized MOF_MIL-101(Fe)_ as the core, embedded NaHCO_3_ as a pH-triggered air bag, electrostatically-adsorbed lentinan forming the inner shell, and a shield shell with 4T1&red blood cell hybrid membrane.

**Results:** HM/Ef/LNT-MOF_MIL-101(Fe)_ can mitigate non-specific capture in the bloodstream but respond to the acidic tumor milieu, rapidly generating a burst of CO_2_ bubbles to disassemble MOF_MIL-101(Fe)_. Upon entering tumors, lentinan-induced interferon-γ (IFN-γ) enable Fe^2+/3+^ facilitating an enhanced ferroptosis and Fenton-like reaction, pushing TAMs towards M1 polarization via the “IFN-γ-ferroptosis-ROS-Caspase-3” pathway. Moreover, HM/Ef/LNT-MOF_MIL-101(Fe)_ increases the infiltration of T lymphocytes and decreases regulatory T cells. These cascading immune responses synergistically foster a loop of amplified TME activation based on TAMs M1 polarization, showcasing notable advancements in anticancer effectiveness and promise for various combination therapies.

**Conclusion:** This study utilizes an "embedded air-bag" strategy to achieve strategic codelivery of Fe^2+/3+^ and lentinan, providing a new tool for engineering the TME.

## Introduction

Iron-based nanocarriers have garnered considerable attention as clinically translatable biomaterials in reorienting the polarization of tumor-associated macrophages (TAMs) towards the M1 phenotype 1, 2. Driven by a series of iron ion-mediated reactions within the tumor microenvironment (TME), iron-based nanocarriers induce ferroptosis 3-5, a non-apoptotic pathway that distinct from classic apoptotic approach, profoundly influencing the TME in the fight against cancer 6. Ferroptosis is primarily characterized by intracellular iron overload, leading to the inhibition of glutathione (GSH) synthesis and a consequent reduction in glutathione peroxidase-4 (GPX-4) activity. This chain of events results in the buildup of lipid peroxides (LPO) and extensive oxidative damage 7. These processes trigger M1 repolarization through releasing tumor-associated antigens (TAAs) 8 and secretion of Th1-type cytokines 9. In the ROS-enriched milieu of the TME, iron-based nanocarriers can also stimulate the Fenton reaction, which reprograms TAMs towards M1 polarization via the "ROS-Caspase-3" pathway 10. This action mitigates regulatory T cell infiltration through the (C-C motif) receptor (CCR) and enhances the antigen presentation of dendritic cells (DCs), thereby activating the entire TME 11, 12. The immunomodulatory effectiveness of iron-based nanocarriers is closely linked to the delivery of iron ions at the tumor site. However, in practical applications, two critical scientific challenges urgently need to be addressed: the insufficient delivery of iron ions and their poor immunomodulatory efficacy. Overcoming the issue of non-specific uptake by the reticuloendothelial system (RES) 13, ensuring the targeted delivery of iron ions to tumors 14, and identifying a suitable synergist are essential steps to resolve these significant obstacles.

Lentinan (LNT), a plant-derived polysaccharide extracted from edible fungi, has been approved by Chinese Food and Drug Administration for boosting the immune response in cancer patients undergoing chemotherapy 15, 16. Although substantial clinical evidence validates its immunomodulatory effects, the specific mechanisms remain unclear. Limited data demonstrated that LNT induced T cells to secrete interferon-γ, inhibiting angiogenesis in 4T1 tumors and facilitating the transition of breast cancer TME from a "cold" to a "hot" state 17. LNT stimulated macrophages to secrete the cytokine IL-12, which in turn induced T cells to produce TNF-γ, thereby achieving direct tumor-killing effects 18. Particularly in the context of triple-negative breast cancer (TNBC), which is characterized by extensive intra-tumoral heterogeneity, lack of biomarkers, and resistance to existing therapies, effective activation of the TME is of paramount importance for augmenting the limited treatment options available 19. However, research on LNT has predominantly focused on T cells, with less emphasis on its interaction with various immune cells within the TME. Recently, an innovative approach involving the delivery of IFN-γ into tumors have shown promising results, such as accelerated depletion of GSH, triggering of ferroptosis, and consequent M1 polarization of TAMs, leading to increased T cell infiltration and thus creating a positive feedback loop for TME activation 20. Therefore, we hypothesize that LNT could synergistically activate the TME in the TNBC with iron-based nanocarriers. This hypothesis relies on efficient delivery to tumor sites, designing a system that enables both LNT and iron-based nanocarriers to circumvent nonspecific uptake by the RES and effectively reach tumor sites is a crucial yet challenging task.

Biomimetic technology based on autologous cell membrane camouflage endows nanomedicines with exceptional immune evasion and targeted delivery 21. These biomimetic nanomedicines not only retain the physicochemical properties of the particles but also inherit the biological recognition and chemotactic characteristics of the cell membranes. Platelet or red blood cell membranes-coated nanoparticles emit a "don't eat me" signal during blood circulation to reduce undesired capture by the RES as possessing CD47 protein 22. Coating with tumor cell membrane enable nanoparticles homotypic targeting through tagging TAAs 23. Nanoparticles enveloped with macrophage, neutrophil, or stem cell membranes reduce uptake by the RES while enhancing homing capabilities 24, 25. Recently, hybrid cell membrane camouflaging amalgamates biological properties from various cell membranes, which has outperformed single cell membrane coatings 26, 27. This approach has demonstrated enhanced efficacy in prolonged blood circulation, antigen recognition, target specificity, and facilitating desired cellular interactions 27. However, membrane coatings may also impede the drug release. Bubble-driven drug release mechanisms, known to create effervescence-like effects, have emerged as promising solutions to overcome these barriers. Our group has harnessed the rapid generation of CO_2_ bubbles induced by NaHCO_3_ in tumors 28, leading to significantly improved drug release and penetration. This strategy is particularly promising for achieving controlled drug release in membrane-camouflaged nanoparticles.

**Scheme 1:**
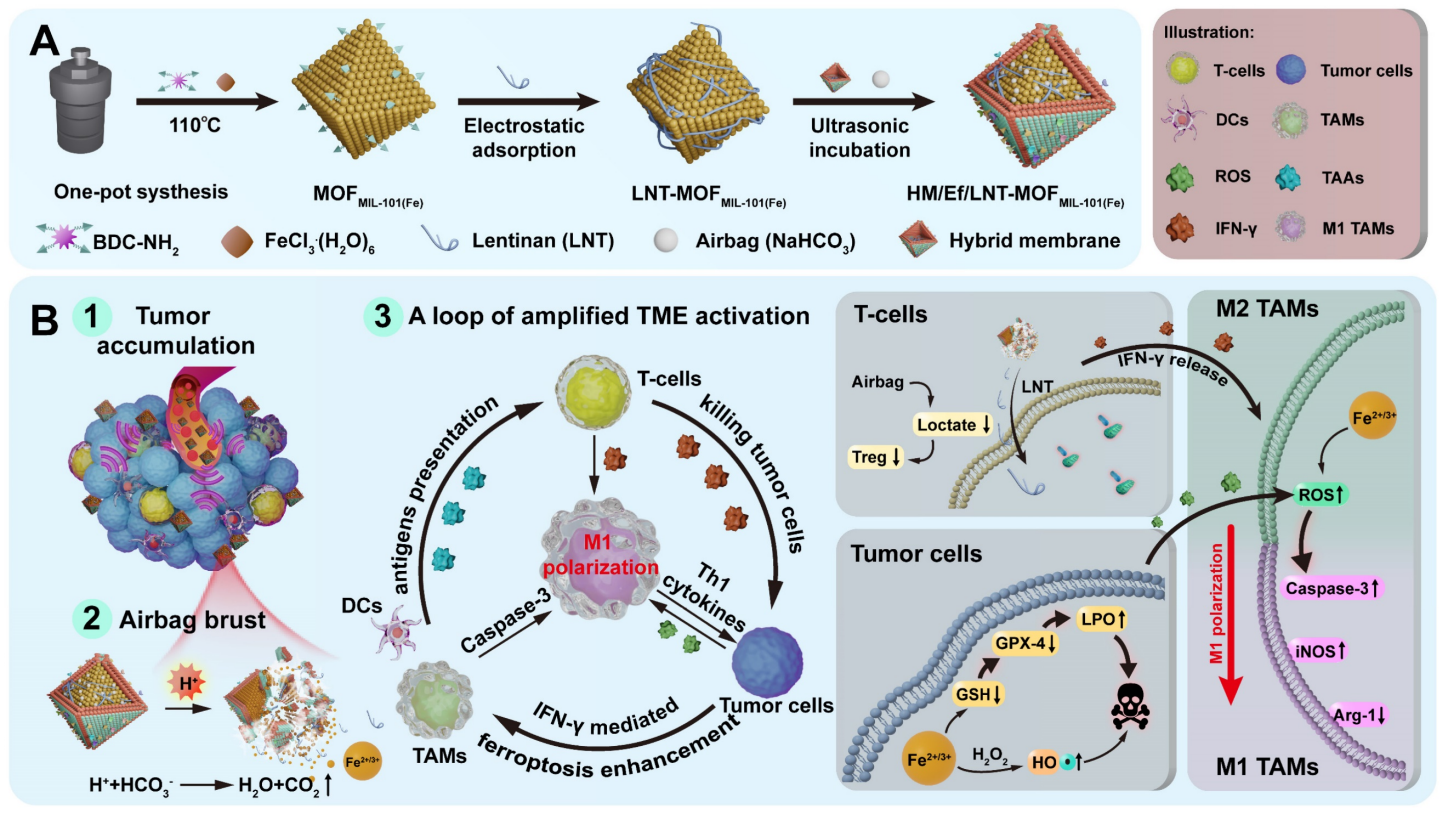
(A) Schematic illustration of preparation of HM/Ef/LNT-MOF_MIL-101(Fe)_. (B) A loop of amplified TME activation centered on TAMs M1 polarization.

MIL-101(Fe) metal-organic frameworks (MOF_MIL-101(Fe)_), a well-known porous crystalline material with an octahedral structure, presents as a promising vector as its ability to drug loading on the basis of the host-guest interactions within the pores and specific surface charge 29-31. Importantly, we found that MOF_MIL-101(Fe)_ can dissociate rapidly under acidic conditions and release iron ions because the coordination between amino benzene dicarboxylate (BDC-NH_2_) linkers and iron significantly diminished. As designed in Scheme 1A, MOF_MIL-101(Fe)_ is employed as a pH-triggered iron-based nanocarrier in this study. NaHCO_3_ serves as an acid-responsive air bag (effervescent component), with LNT electrostatically attached as an inner shell layer. The outer layer is coated with a hybrid membrane of breast cancer 4T1 cells and red blood cells, fabricating HM/Ef/LNT-MOF_MIL-101(Fe)_. This system circumvents non-specific capture by the RES during the circulation, accumulates at tumor sites through homotypic targeting, and activates the air bag to accelerate the dissociation of each component. As illustrated in Scheme 1B, this process facilitates the rupture of both the hybrid cell membrane and intermediate LNT layer, enabling rapid release of therapeutics at tumor sites. Upon entering tumors, LNT induces T cells to release IFN-γ, fostering an enhanced ferroptosis via the “IFN-γ-GSH-GPX4-LPO” pathway. Tumor cells undergoing ferroptosis and the Fenton reaction reprogram TAMs towards M1 polarization through the “ROS-Caspase-3” pathway. Ferroptosis also can promote the mature of DCs. Moreover, LNT increases the infiltration of T cells by normalizing mitochondrial function, meanwhile, air bag-mediated lactate consumption decreases regulatory T cell populations. These cascading reactions foster a loop of amplified TME activation on the basis of TAMs M1 polarization, revitalizing various therapeutic strategies for TNBC treatment. This study will focus on the preparation and characterizations of HM/Ef/LNT-MOF_MIL-101(Fe)_, *in vivo* and *in vitro* TME activation, and anti-tumor activity evaluation. This study leverages an "embedded air-bag" strategy to achieve strategic codelivery of iron ions and LNT, providing new perspectives and methodologies for engineering the TME.

## Results and Discussion

### Preparation and characterizations

**Figure 1:**
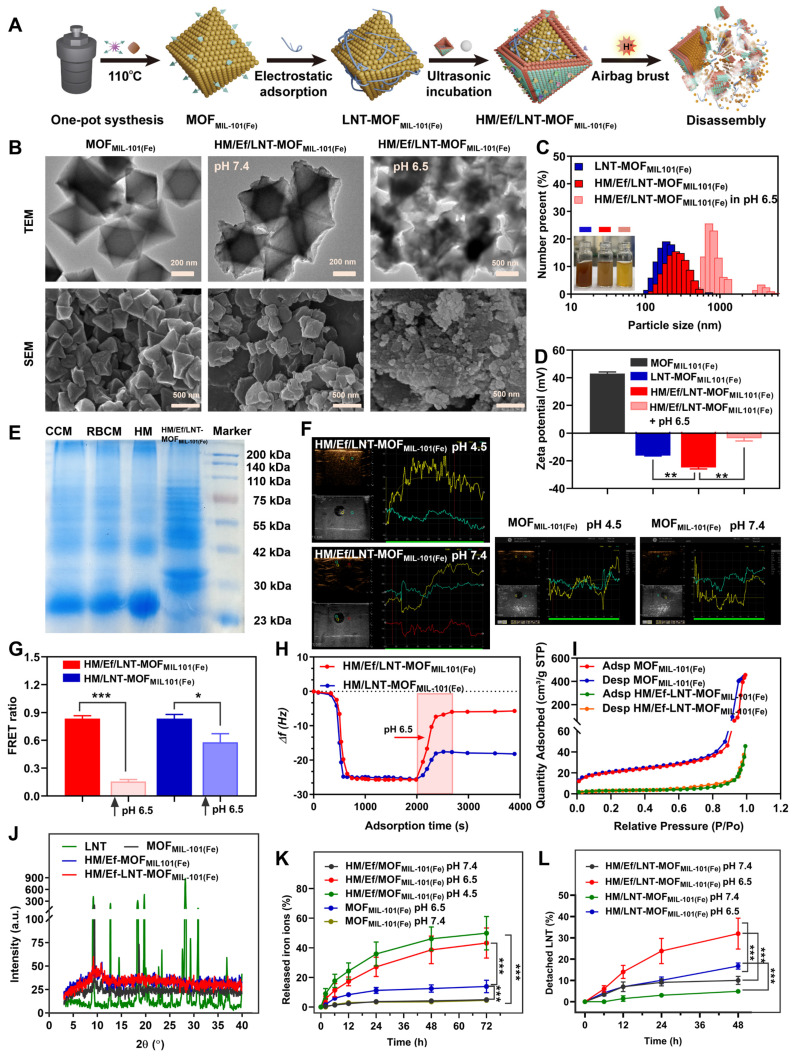
Preparation and characterizations of HM/Ef/LNT-MOF_MIL-101(Fe)._ (A) Schematic illustration of preparation and disassembly of HM/Ef/LNT-MOF_MIL-101(Fe)._ (B) TEM and SEM images of HM/Ef/LNT-MOF_MIL-101(Fe)_ at different pH environments. (C) Particle size and (D) zeta potential studied by DLS. Data are represented as mean ± SD; n = 5. **p < 0.01. The inserted pictures are aqueous solution of corresponding sample. (E) The protein bands of membranes and HM/Ef/LNT-MOF_MIL-101(Fe)_ evaluated with SDS-PAGE. CCM and RBCM represent cancer cell membrane and red blood cell membrane, respectively. (F) Ultrasound imaging of bubbles in agarose gel. (G) FRET ratios of fluorescence-labelled samples at different pH environments. Data are represented as mean ± SD; n = 4. *p < 0.05, ***p < 0.001. (H) Changes in mass signal upon stimulation of pH 6.5 studied by QCM. (I) Nitrogen adsorption and desorption isotherms of various formulations. (J) XRD patterns of different formulations. Release profiles of (K) iron ions and (L) LNT of different formulations at various pH values. Data are represented as mean ± SD; n = 4. ***p < 0.001.

The fabrication process of HM/Ef/LNT-MOF_MIL-101(Fe)_ is outlined in Figure 1A. The process began with the solvothermal synthesis of MOF_MIL-101(Fe)_ using BDC-NH_2_ and FeCl_3_.6H_2_O, forming an octahedral structure. Subsequently, LNT was electrostatically adsorbed to obtain LNT-MOF_MIL-101(Fe)_. NaHCO_3_ and a hybrid membrane were ultrasonically incubated with LNT-MOF_MIL-101(Fe)_ to gain HM/Ef/LNT-MOF_MIL-101(Fe)_. As shown in Table S1, the drug loading (DL) of LNT and NaHCO_3_ in HM/Ef/LNT-MOF_MIL-101(Fe)_ were 3.1 ± 0.3% and 14.5 ± 2.4%, respectively. The weight percentage of iron ions and proteins in HM/Ef/LNT-MOF_MIL-101(Fe)_ were 4.6 ± 0.3% and 23.5 ± 2.1%, respectively. Comparisons among various groups indicate that neither the hybrid membrane coatings nor airbag loading significantly affect the DL of each component. As shown in Figure 1B, the TEM image of MOF_MIL-101(Fe)_ exhibited an octahedral structure with a size of approximately 250 nm. In contrast, the outer layer of HM/Ef/LNT-MOF_MIL-101(Fe)_ had a clear coating with a membrane-like structure while retaining the underlying octahedral backbone. Notably, upon exposure to pH 6.5, significant structure disassembly was observed in the HM/Ef/LNT-MOF_MIL-101(Fe)_ group, aligning with SEM characterizations. As demonstrated in Figure 1C, the hybrid membrane encapsulation remarkably improved the water solubility of MOF_MIL-101(Fe)_. HM/Ef/LNT-MOF_MIL-101(Fe)_ solution became transparent and reddish-brown at pH 6.5, indicating potential bubble-driven disassembly and subsequent release of therapeutics. The DLS results showed that HM/Ef/LNT-MOF_MIL-101(Fe)_ had a particle size of approximately 250 nm, which sharply increased under pH 6.5 stimulation (Figures 1C). Similarly, the zeta potential of -25 mV moved towards neutrality (Figures 1D), again suggesting potential electrostatic adsorption and disassembly.

Sodium dodecyl sulfate-polyacrylamide gel electrophoresis (SDS-PAGE) demonstrates that the protein bands of the hybrid membrane were similar with those of HM/Ef/LNT-MOF_MIL-101(Fe)_ (Figure 1E). The ultrasound imaging presented that HM/Ef/LNT-MOF_MIL-101(Fe)_ could rapidly generate a large number of bubbles in pH 4.5 (Figure 1F), whereas MOF_MIL-101(Fe)_ itself did not produce bubbles, suggesting that the embedded airbag is responsive to the acidic environment. In Figure 1G, we used the FRET technique to verify the dissociation of HM/Ef/LNT-MOF_MIL-101(Fe)_ under weak acidic environment. FITC and DiI were here employed as a FRET fluorescence pair to label the LNT and hybrid membrane in HM/Ef/LNT-MOF_MIL-101(Fe)_, respectively. When the structure was stable, a high FRET ratio (~ 0.84 ± 0.04) was observed due to the proximity of the hybrid membrane and LNT. Upon acidic stimulation, the FRET ratio drastically decreased (~ 0.15 ± 0.01), suggesting a detachment of the hybrid membrane and LNT. The QCM measurement in Figure 1H presented a significant mass loss (81.5%) for HM/Ef/LNT-MOF_MIL-101(Fe)_ under acidic stimulation, with most components dissociation except for the hybrid membrane bound to the QCM chip. In contrast, only 28.3% of weight loss was detected in HM/LNT-MOF_MIL-101(Fe)_, suggesting a suppression of disassembly in absence of bubble flow.

As depicted in Figure 1I, the N_2_ adsorption volume of HM/Ef/LNT-MOF_MIL-101(Fe)_ was 43.4 cm^3^/g STP, significantly lower than that of MOF_MIL-101(Fe)_, indicating that therapeutic agents probably fill the pores of MOF_MIL-101(Fe)_. The XRD results in Figure 1J demonstrated that HM/Ef/LNT-MOF_MIL-101(Fe)_ retained the crystalline characteristics of MOF_MIL-101(Fe)_, but that of surface-adsorbed LNT disappeared, suggesting an amorphous incorporation of LNT within the particles.

As the release of iron ions and LNT examined in Figures 1K and 1L, HM/Ef/LNT-MOF_MIL-101(Fe)_ released 43.2 ± 6.5% of iron ions under pH 6.5, significantly higher than MOF_MIL-101(Fe)_, and over 31.9 ± 7.9% of LNT, remarkably outperforming HM/LNT-MOF_MIL-101(Fe)_. Notably, the iron release profiles of the two formulations at pH 7.4 are very close to each other. These results further underline the critical role of airbag design for the controlled release of therapeutics.

### Cellular uptake and intracellular delivery

**Figure 2:**
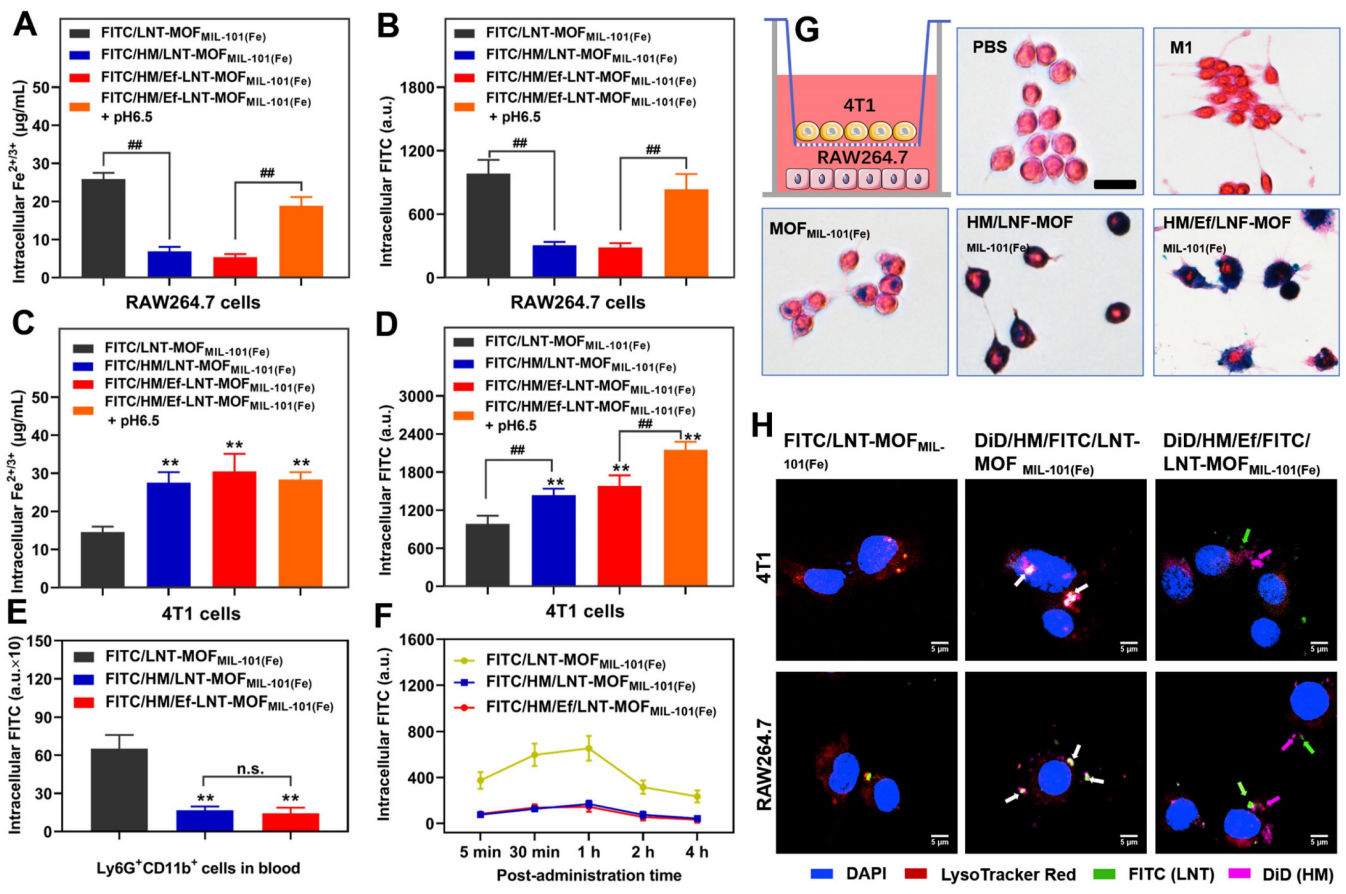
Intracellular uptake and delivery. Intracellular uptake of (A) Fe^2+/3+^ and (B) FITC in RAW264.7 cells after treatments. Intracellular uptake of (C) Fe^2+/3+^ and (D) FITC in 4T1 cells after treatments. Intracellular uptake of (E) FITC from Ly6G^+^CD11b^+^ cells in blood of mice treated with different formulations. Data are presented as mean ± SD; n = 3. **p < 0.01 vs. FITC/LNT-MOF_MIL-101(Fe)_; ^##^*p* < 0.01. (F) Dynamic uptake by Ly6G^+^CD11b^+^ cells after different treatments within 4 h. (G) Prussian blue staining of RAW264.7 cells when co-incubated with 4T1 cells using Transwell technology. (H) Intracellular delivery of various formulations in 4T1 cells and RAW264.7 cells. The red, green, and pink fluorescence label lysosomes, LNT, and HM, respectively. White arrows indicate the overlap of the three fluorescence channels, while green and pink arrows represent the disassembly of HM/Ef/LNT-MOF_MIL-101(Fe)_ and its escape from lysosomes. The scale bar is 5 μm.

The aim of this section is to assess the impact of hybrid membrane encapsulation and airbag design on the cellular uptake of HM/Ef/LNT-MOF_MIL-101(Fe)_ across various cell lines. Here, FITC was utilized to label the LNT, enabling precise quantitative analysis. As revealed in Figures 2A and 2B, the cellular uptake of iron ions and LNT by RAW264.7 cells were significantly reduced following hybrid membrane modification; however, the internalization markedly improved after stimulation with pH 6.5, likely due to disassembly within the acidic milieu. In contrast, the hybrid membrane-modified groups showed a substantial enhancement in 4T1 cellular uptake of both iron ions and LNT compared to the group treated with FITC/LNT-MOF_MIL-101(Fe)_ (Figures 2C and 2D).

During blood circulation, the FITC signal in Ly6G^+^CD11b^+^ cell populations (neutrophils) 32 were significantly lower in the hybrid membrane-coated groups compared to FITC/LNT-MOF_MIL-101(Fe)_ group (Figure 2E), underscoring the effectiveness of hybrid membrane encapsulation in reducing non-specific uptake by blood-circulating neutrophils. Despite the equipment with an airbag design, the neutrophil uptake of FITC/HM/Ef/LNT-MOF_MIL-101(Fe)_ did not show a significant increase, suggesting the potential stability of the HM/Ef/LNT-MOF_MIL-101(Fe)_ structure in blood circulation. During the 4 h post-treatment period, FITC/HM/Ef/LNT-MOF_MIL-101(Fe)_ consistently exhibited a low level of non-specific neutrophil capture (Figure 2F), demonstrating a clear advantage over the FITC/LNT-MOF_MIL-101(Fe)_ group.

Further, as Prussian blue staining results illustrated in Figure 2G, when HM/Ef/LNT-MOF_MIL-101(Fe)_ was applied to 4T1 cells in the upper chamber, it significantly induced dendritic growth of RAW264.7 cells in the lower chamber. This was accompanied by a notable increase in iron uptake, pointing to a potential M1 polarization of macrophages driven by enhanced ferroptosis.

The intracellular delivery results demonstrate that FITC/DiD-dual labeled HM/LNT-MOF_MIL-101(Fe)_ might be localized within the endo-lysosomes of both 4T1 cells and RAW264.7 cells, as evidenced by the overlapping fluorescence signals. In contrast, treatment with FITC/DiD-dual labeled HM/Ef/LNT-MOF_MIL-101(Fe)_ resulted in distinct green, pink, and red fluorescence signals observed within the cytoplasm (Figure 2H), indicative of acid-mediated disassembly and lysosomal escape. These behaviors are highly associated with the pH-triggered airbag design.

### Polarization of macrophages* in vitro*

**Figure 3:**
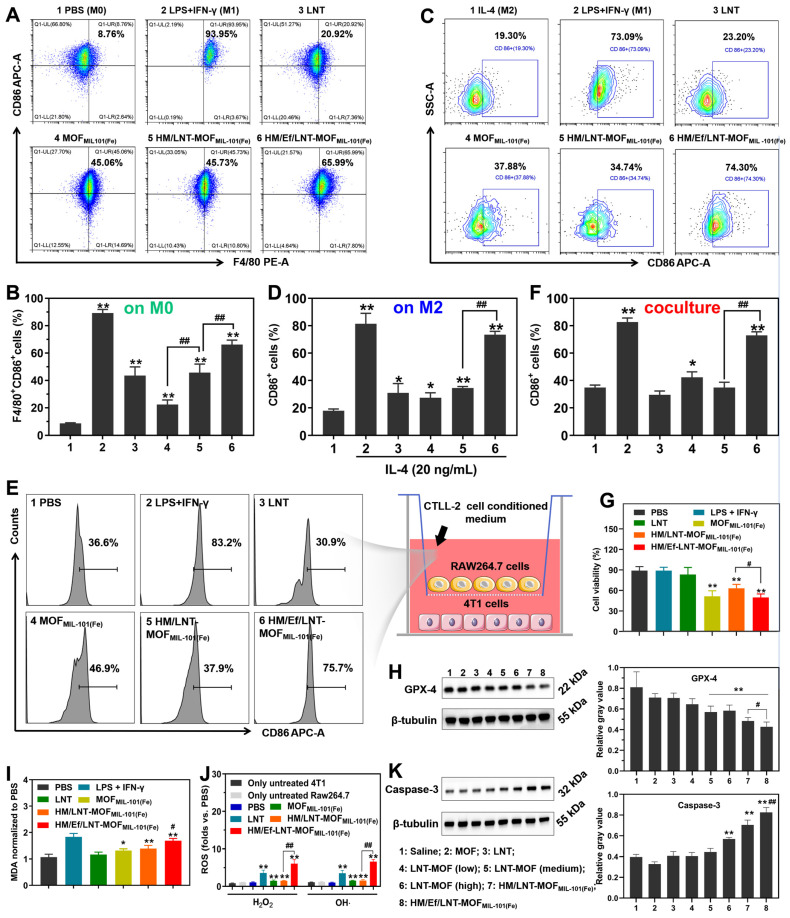
Polarization assays *in vitro.* (A) Flow cytometry analysis and (B) quantification of CD86 expression in naïve macrophages. Data are presented as mean ± SD; n = 3. **p < 0.01 vs. PBS; ^##^*p* < 0.01. (C) Flow cytometry analysis and (D) quantification of CD86 expression in IL-4-pretreated macrophages. Data are presented as mean ± SD; n = 3. *p < 0.05, ^**^P < 0.01 vs. IL-4; ^##^*p* < 0.01. (E) Flow cytometry analysis and (F) quantification of CD86 expression in RAW264.7-4T1 cell coculture model. (G) Cytotoxicity of 4T1 cells in cell coculture model. Data are presented as mean ± SD; n = 3. *p < 0.05, **p < 0.01 vs. PBS; ^#^*p* < 0.05, ^##^*p* < 0.01. (H) Western blot analysis of GPX-4 in 4T1 cells after being treated with different formulations. Data are presented as mean ± SD; n = 3. **p < 0.01 vs. Saline; ^#^*p* < 0.05 vs. HM/LNT-MOF_MIL-101(Fe)_. (I) Relative MDA and (J) ROS in 4T1 cells after treated with different formulations. Data are presented as mean ± SD; n = 3. *p < 0.05, **p < 0.01 vs. PBS;^ #^*p* < 0.05, ^##^*p* < 0.01 vs. HM/LNT-MOF_MIL-101(Fe)_. (K) Western blot analysis of Caspse-3 in RAW264.7 cells after being treated with different formulations. Data are presented as mean ± SD; n = 3. **p < 0.01 vs. Saline; ^##^*p* < 0.01 vs. HM/LNT-MOF_MIL-101(Fe)_. Results in (E ~ K) were obtained from cell coculture model.

In this section, the M1 polarization of macrophages by HM/Ef/LNT-MOF_MIL-101(Fe)_ in M0/M2 RAW264.7 cell and RAW 264.7-4T1 cell coculture models were investigated. As demonstrated in Figures 3A and 3B, both LNT and MOF_MIL-101(Fe)_ significantly enhanced CD86 expression in naive (M0) macrophages. HM/Ef/LNT-MOF_MIL-101(Fe)_ induced greater M1 polarization in macrophages compared to MOF_MIL-101(Fe)_ and surpassed the performance of HM/LNT-MOF_MIL-101(Fe)_. This indicates that the rapid release of therapeutics triggered by the airbag design is beneficial to M1 polarization. In IL-4-pretreated (M2) macrophage models, increased CD86 expression was still observed in both the LNT and MOF_MIL-101(Fe)_ groups. As expected, treatment with HM/Ef/LNT-MOF_MIL-101(Fe)_ resulted in significantly higher CD86 expression compared to the non-airbag design (Figures 3C and 3D).

To investigate the potential role of LNT in the TAMs M1 polarization, a transwell coculture model was constructed with RAW264.7 cells in the upper chamber and 4T1 cells in the lower chamber. The medium employed the conditioned medium from coculture with LNT and CTLL-2 cells for 48 h (Figure 3E). As shown in Figure S1, CTLL-2 cells exhibited a 1.5-fold of IFN-γ secretion after treatment with the LNT, thereby creating a high IFN-γ environment for the coculture model. As shown in Figures 3E and 3F, MOF_MIL-101(Fe)_ led to a CD86^+^ cell percentage of 42.3 ± 4.4%, significantly exceeding that of the PBS group. Notably, after treatment with HM/Ef/LNT-MOF_MIL-101(Fe)_, the proportion of CD86^+^ cells surged to 74.3 ± 2.1%, markedly higher than the HM/LNT-MOF_MIL-101(Fe)_ group. Additionally, the M1-polarized RAW264.7 cells by HM/Ef/LNT-MOF_MIL-101(Fe)_ significantly reduced the viability of 4T1 cells to 49.3% (Figure 3G), notably lower than HM/LNT-MOF_MIL-101(Fe)_ group. The viability of HM/Ef/LNT-MOF_MIL-101(Fe)_ against RAW264.7 cells and 4T1 cells were 98.3 ± 6.8% and 77.2 ± 3.9%, respectively (Figure S2). Therefore, we proposed a hypothesis that such M1 polarization triggers cytotoxicity against 4T1 cells are primarily due to HM/Ef/LNT-MOF_MIL-101(Fe)_ enhances ferroptosis through LNT-mediated IFN-γ generation, with the airbag design contributing as a crucial factor.

Next, GPX-4 expression and MDA secretion were examined, which are classic markers of ferroptosis 33. As demonstrated in Figures 3H and 3I, GPX-4 expression in HM/Ef/LNT-MOF_MIL-101(Fe)_-treated 4T1 cells was notably lower than in the HM/LNT-MOF_MIL-101(Fe)_-treated group, while MDA secretion increased significantly, suggesting enhanced ferroptosis. Besides, Figure 3J illustrated that the generation of hydroxyl radicals in the HM/Ef/LNT-MOF_MIL-101(Fe)_ group was 7.1 times higher than in the PBS group, also significantly exceeding that in both HM/LNT-MOF_MIL-101(Fe)_ and MOF_MIL-101(Fe)_ groups, suggesting a more efficient Fenton reaction. The increased H_2_O_2_ and OH· led to elevated Caspase-3 activity (Figure 3K), considered a key factor in inducing M1 polarization in macrophages 10.

### Tumor accumulation *in vivo*

**Figure 4:**
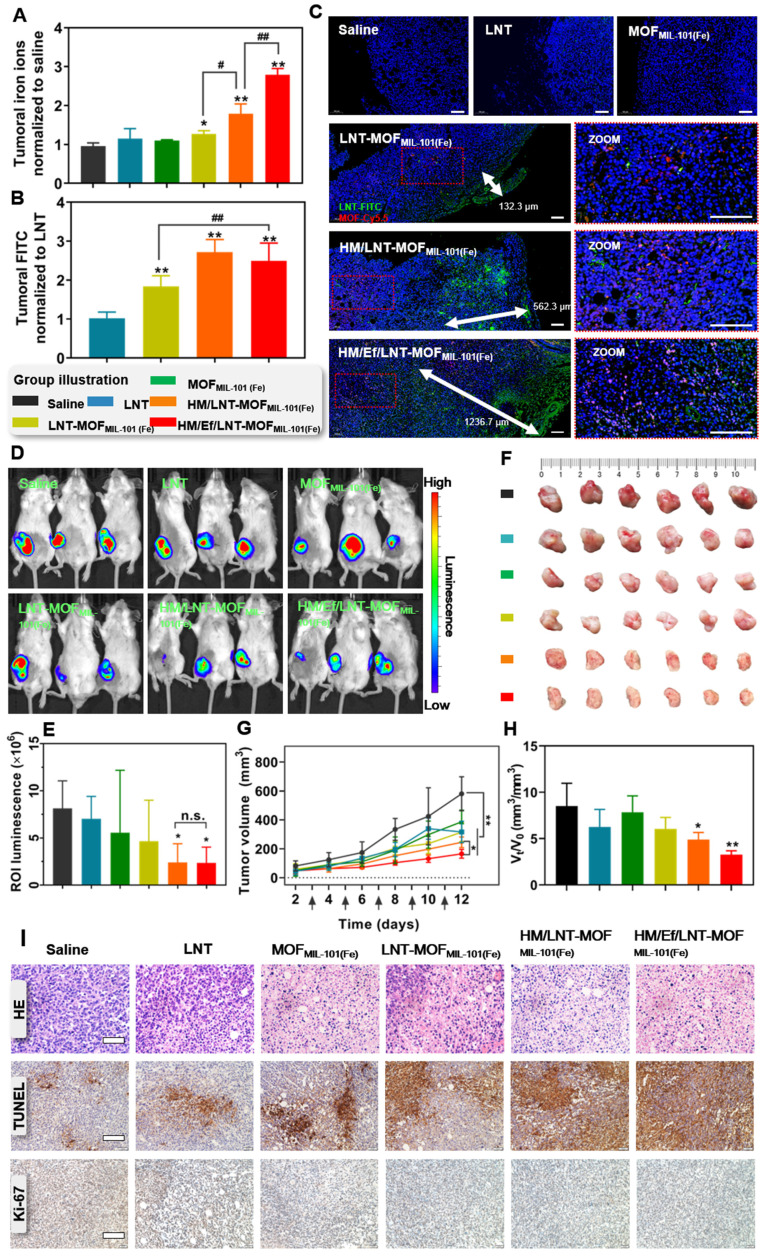
Biodistribution and anticancer efficacy *in vivo*. Relative (A) iron ions and (B) LNT in tumors after treatments with various formulations. The data are shown as mean ± SD, n = 3, *p < 0.05; **p < 0.01 vs. Saline; ^#^*p* < 0.05, ^##^*p* < 0.01. (C) Fluorescence-stained tumor sections after treatments. The scale bar is 100 μm. (D) Bioluminescence images and (E) region-of-interest luminescence of orthotopic Luc-4T1 tumor-bearing mice at d11 post-implantation. The data are shown as mean ± SD, n = 3, *p < 0.05 vs. Saline. (F) The images of *ex vivo* tumor at the end of the treatments. (G) Tumor growth curve of each mouse during the treatments. The arrows present administration time. The data are shown as mean ± SD, n = 6, *p < 0.05; **p < 0.01. (H) Ratio of tumor volume at day 11 to day 1 (V_t_/V_0_). The data are shown as mean ± SD, n = 6, ^*^p < 0.05; **p < 0.01 vs. Saline. (I) HE staining, TUNEL immunofluorescence, and Ki-67 immunohistochemistry images of tumor sections after the treatments. The scale bars are 100 μm, 200 μm, and 200 μm, respectively.

In this part, both qualitative and quantitative analysis of the intratumoral accumulation and distribution of LNT and iron ions were investigated to verify the precise delivery *in vivo*. As shown in Figure 4A, the concentration of intratumoral iron ions in the LNT-MOF_MIL-101(Fe)_ treatment group was 30.4% higher than in the saline group. Notably, HM/LNT-MOF_MIL-101(Fe)_ enhanced intratumoral iron ion accumulation due to the hybrid membrane encapsulation. With a similar coating, HM/Ef/LNT-MOF_MIL-101(Fe)_ displayed a significantly higher accumulation of iron ions than the HM/LNT-MOF_MIL-101(Fe)_ group, reaching 2.8 times that of the saline group, which might be attributed to the airbag-triggered rapid disassembly within the tumor milieu. However, the airbag-mediated effervescent effect had minimal impact on LNT tumor accumulation. The LNT accumulation in HM/Ef/LNT-MOF_MIL-101(Fe)_ was 2.3 times greater than that of free LNT (Figure 4B), providing sufficient exposure for enhanced iron ion-mediated ferroptosis.

As shown in Figure S3, the distribution of iron ions in various tissues at 6 h and 24 h post-administration was detected. At 6 h after treated with HM/Ef/LNT-MOF_MIL-101(Fe)_, the iron ions in the tumor were 13.62 ± 3.66 ID%, which is only lower than that in the liver and significantly higher than in other tissues. At 24 h post treatments, the iron ions in the tumor were 9.52 ± 2.21 ID%, which is the highest among all tissues. As demonstrated in Figure 4C, tumor sections labeled with Cy5.5 (red) and FITC (green) represented MOF and LNT, respectively. Both red and green fluorescence in the HM/Ef/LNT-MOF_MIL-101(Fe)_ and HM/LNT-MOF_MIL-101(Fe)_ groups were significantly stronger than in the LNT-MOF_MIL-101(Fe)_ group, emphasizing the importance of hybrid membrane modification for tumor accumulation. The rapid disassembly induced by the CO_2_ bubble flow in the HM/Ef/LNT-MOF_MIL-101(Fe)_ group resulted in a green fluorescence penetration depth of 1236.7 μm, surpassing that of groups without an airbag design. These finds indicate that the hybrid membrane coating and airbag loading contribute to the desired tumoral delivery of iron ions and LNT.

### Antitumor efficacy *in vivo*

In this part, we evaluated the impact of improved co-delivery to tumors on anticancer efficacy. As illustrated in Figures 4D and 4E, mice bearing Luc-4T1 breast cancer tumors treated with HM/Ef/LNT-MOF_MIL-101(Fe)_ exhibited significantly reduced luminescence at the tumor sites compared to those treated with saline. Additionally, as depicted in Figure 4F, the *ex vivo* tumors from the HM/Ef/LNT-MOF_MIL-101(Fe)_ group at the end of the treatment period displayed the smallest size among all the groups. The progression of tumor growth, as shown in Figure 4G, indicated that the HM/Ef/LNT-MOF_MIL-101(Fe)_ group experienced the most pronounced suppression of tumor growth, with a tumor inhibition rate of 48.2 ± 5.3%, which is notably higher than the HM/LNT-MOF_MIL-101(Fe)_ group (36.5 ± 4.2%). Furthermore, the tumor growth rate, assessed by the V_t_/V_0_ ratio, was significantly lower in the HM/Ef/LNT-MOF_MIL-101(Fe)_ group at 3.79 compared to all other treatment groups (Figure 4H). Examination of tumor slices, as depicted in Figure 4I, revealed pronounced tissue necrosis in the HM/Ef/LNT-MOF_MIL-101(Fe)_ group, characterized by a substantial spread of TUNEL-positive areas and the least intense Ki-67 positive signal. These observations suggest that tumors in the HM/Ef/LNT-MOF_MIL-101(Fe)_ group undergo extensive necrosis and apoptosis, leading to a significant reduction in their proliferation following the treatment 34.

### Influence on TAMs

**Figure 5:**
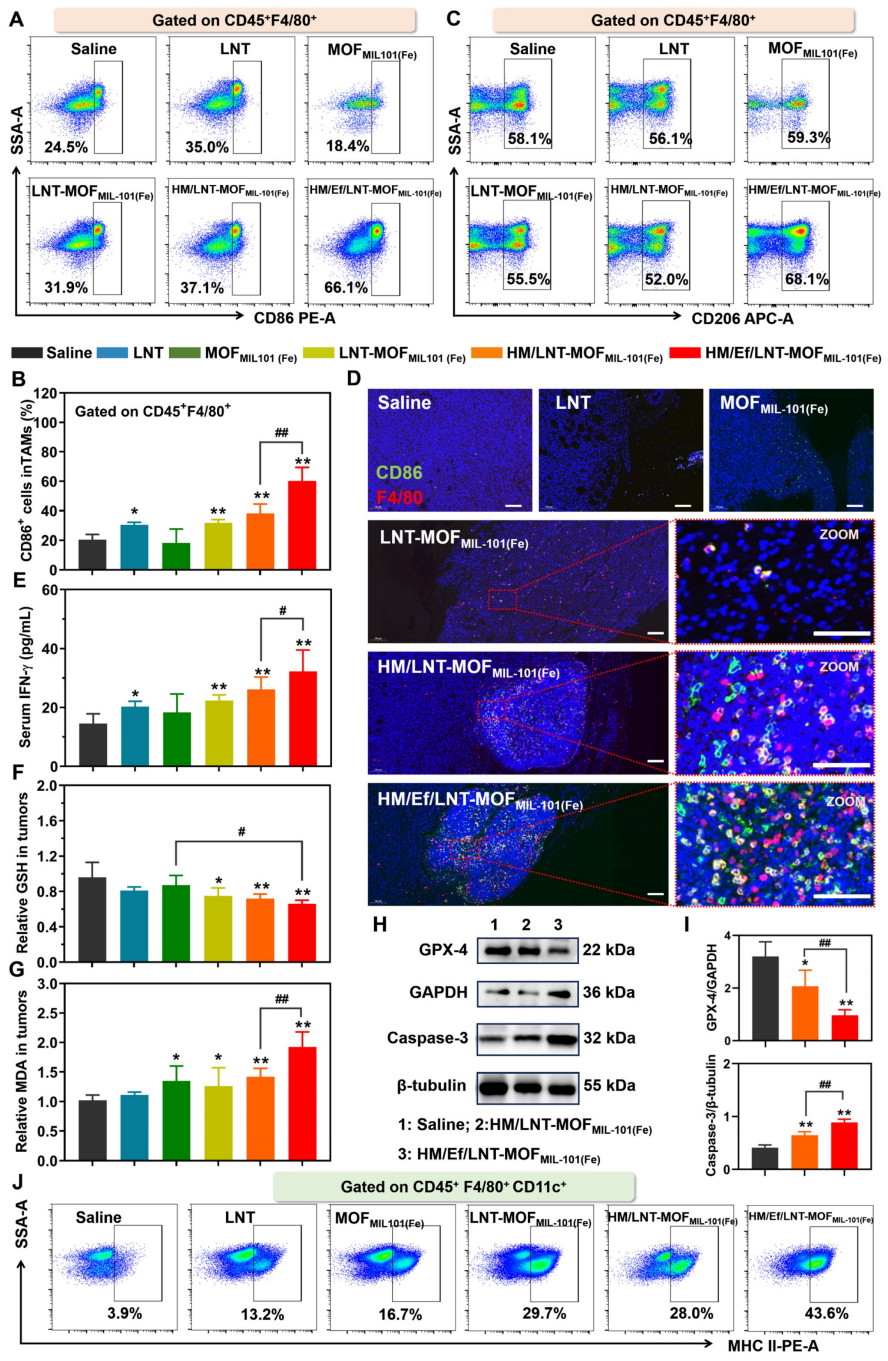
TAMs polarization and ferroptosis *in vivo*. Flow cytometer analysis of (A ~ B) CD86 and (C) CD206 expression in TAMs gated on CD45^+^F4/80^+^ cell populations. (D) F4/80&CD86 dual-stained immunofluorescence images of tumor sections. The bar is 100 μm. (E) Serum INF-γ of mice treated with different formulations. (F) GSH and (G) MDA content in tumors of mice after different treatments. Western blot analysis for (H) GPX-4 and Caspase-3 from tumor-derived protein and (I) the quantification of gray value. (J) Flow cytometer analysis of MHC-II expression in TAMs gated on CD45^+^F4/80^+^CD11c^+^ cell populations. The data are shown as mean ± SD, n = 3, *p < 0.05, **p < 0.01 vs. Saline; ^#^*p* < 0.05,^ ##^*p* < 0.01.

As illustrated in Figures 5A and 5B, M1 TAMs constituted a minority in the TME of saline-treated mice bearing 4T1 tumors. LNT treatment induced TAMs repolarize towards the M1 phenotype, while the blank MOF_MIL-101(Fe)_ exhibited negligible similar effects. Neither the LNT-MOF_MIL-101(Fe)_ nor HM/LNT-MOF_MIL-101(Fe)_ groups demonstrated significant enhancement compared to LNT alone, suggesting suboptimal co-delivery efficiency of LNT and iron ions to the tumor sites. However, HM/Ef/LNT-MOF_MIL-101(Fe)_, with bubble-mediated disassembly, significantly elevated the M1 TAMs (CD45^+^F4/80^+^CD86^+^ cells) proportion in the TME, a finding validated by both qualitative and quantitative data (Figures 5D). Interestingly, none of the treatments notably affected the M2 TAMs populations (Figures 5C and Figure S4), which is different from the conclusion that M1 TAMs have stronger resistance to ferroptosis than M2 phenotypes 35.

As shown in Figure 5E, all groups containing LNT elevated intratumoral IFN-γ levels, with HM/Ef/LNT-MOF_MIL-101(Fe)_ further increasing it, likely associated with the enhanced LNT exposure at tumor sites. The treatment with HM/Ef/LNT-MOF_MIL-101(Fe)_ significantly increased GSH consumption (Figure 5F) and MDA accumulation (Figure 5G) compared to saline-treated group, meanwhile, while downregulating GPX-4 protein expression (Figure 5H and 5I), indicative of an intensified ferroptosis process. Moreover, HM/Ef/LNT-MOF_MIL-101(Fe)_ treatment fostered an efficient Fenton-like reaction in the H_2_O_2_-rich tumor milieu, probably due to improved delivery of iron ions 36. This results in significantly elevated ROS levels (Figure S5) and enhanced Caspase-3 activity (Figure 5H and 5I). Such mechanism is reported as a primary driver for TAMs M1 polarization by iron ion-based nanocarriers 10, 37. The intensified ferroptosis and Fenton reaction-co-triggered cell death released tumor-associated antigens (TAAs), facilitating dendritic cells (DCs) maturation and antigen presentation. As expected, HM/Ef/LNT-MOF_MIL-101(Fe)_ treatment markedly increased the matured DCs proportion (CD45^+^F4/80^+^CD11c^+^MHCII^+^ populations, Figure 5J), significantly higher than the HM/LNT-MOF_MIL-101(Fe)_ and LNT-MOF_MIL-101(Fe)_ groups (Figure S6). The tumor cells underwent ferroptosis, releasing TAAs, which potentially induced M1 polarization in TAMs and maturation in DCs 38.

In summary, HM/Ef/LNT-MOF_MIL-101(Fe)_ facilitates an amplified ferroptosis in the tumor via the “IFN-γ-GSH-GPX4-LPO” pathway, achieved through LNT-induced INF-γ elevation and enhanced co-delivery of LNT and iron ions. This process is coupled with the ROS generated from the Fenton-like reaction and increased Caspase-3 activity. Consequently, this formulation synergistically enhances the TAMs M1 polarization, which validates the hypothesis proposed in Scheme 1B.

### Influence on T lymphocytes

**Figure 6:**
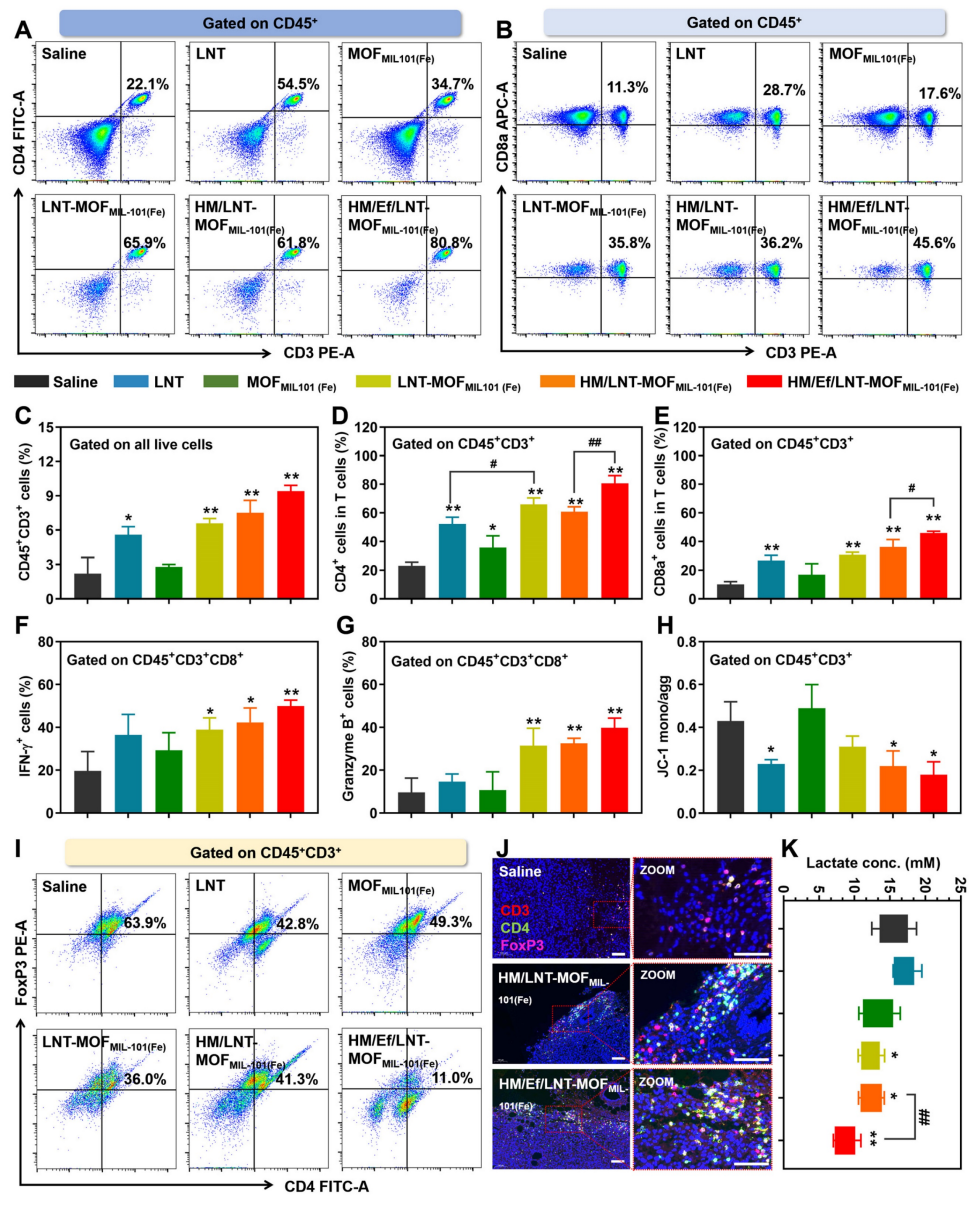
Influence on T cells *in vivo*. Flow cytometer analysis of (A) CD4 and (B) CD8a expression in T cells gated on CD45^+^CD3^+^ cell populations. Percentage of (C) CD45^+^CD3^+^ cells, (D) CD45^+^CD3^+^CD4^+^ cells, and (E) CD45^+^CD3^+^CD8a^+^ cells in the TME. Percentage of (F) INF-γ-positive and (G) Granzyme B-positive cells in CD45^+^CD3^+^CD8a^+^ cell populations after mice were treated with different formulations. (H) Mitochondrial membrane potential of T cells studied by flow cytometry. (I) Flow cytometer analysis of FoxP3 expression in CD4^+^ T cells gated on CD45^+^CD3^+^ cell populations. (J) CD3&CD4&FoxP3 triple-stained immunofluorescence images of tumor sections. The bar is 100 μm. (K) Lactate content in tumors of mice treated with different formulations. The data are shown as mean ± SD, n = 4, *p < 0.05, **p < 0.01 vs. Saline; ^#^*p* < 0.05; ^##^*p* < 0.01.

As evidenced in both clinical and cellular studies, LNT has established efficacy in T cell activation. In this study, we aim to determine whether improved intratumoral delivery of LNT correlates with enhanced T cell activation. As demonstrated in Figure 6A ~ 6E, HM/Ef/LNT-MOF_MIL-101(Fe)_ markedly increased the infiltration of total T cells (CD45^+^CD3^+^ populations) within the TME, along with significant increases in both cytotoxic (CD45^+^CD3^+^CD8a^+^) and helper (CD45^+^CD3^+^CD4^+^) T cell populations. To further assess the vitality of cytotoxic T cells, we also analyzed the expression of IFN-γ and Granzyme B in the CD45^+^CD3^+^CD8a^+^ cell populations. As shown in Figure 6F and 6G, HM/Ef/LNT-MOF_MIL-101(Fe)_-treated tumors exhibited significantly higher proportions of CD8a^+^IFN-γ^+^ and CD8a^+^Granzyme B^+^ cells compared to the saline and HM/LNT-MOF_MIL-101(Fe)_ groups. The roles of the hybrid membrane encapsulation and the air bag design are critical in strengthening T cell vitality, indicating that efficient delivery of the LNT is pivotal for T cell infiltration and activation. Additionally, LNT was observed to maintain the mitochondrial membrane potential in T cells, thus promoting their vitality, while MOF_MIL-101(Fe)_ showed minimal impact on this aspect (Figure 6H).

Given the role of regulatory T (T_reg_) cells in tumor immunosuppression, the influence of HM/Ef/LNT-MOF_MIL-101(Fe)_ treatment on T_reg_ cells was also examined. As exhibited in Figure 6I and 6J, there was a significant reduction in the T_reg_ cell (CD45^+^CD3^+^CD4^+^FoxP3^+^) proportions after HM/Ef/LNT-MOF_MIL-101(Fe)_ treatment, notably lower than in the HM/LNT-MOF_MIL-101(Fe)_ and LNT-MOF_MIL-101(Fe)_ groups. Lactate, pivotal in the TME for T_reg_ cell proliferation and function, was substantially reduced by the HM/Ef/LNT-MOF_MIL-101(Fe)_ treatment through proton consumption facilitated by the airbag design (Figure 6K), thereby disrupting the favorable environment for T_reg_ cells.

In summary, HM/Ef/LNT-MOF_MIL-101(Fe)_ primarily facilitates the infiltration of cytotoxic and effector T cells through LNT, while concurrently suppressing T_reg_ cell proliferation by reducing tumor lactate levels, thus effectively activating the TME from a T cell-centric perspective.

### Influence on cytokines and other immune cells

**Figure 7:**
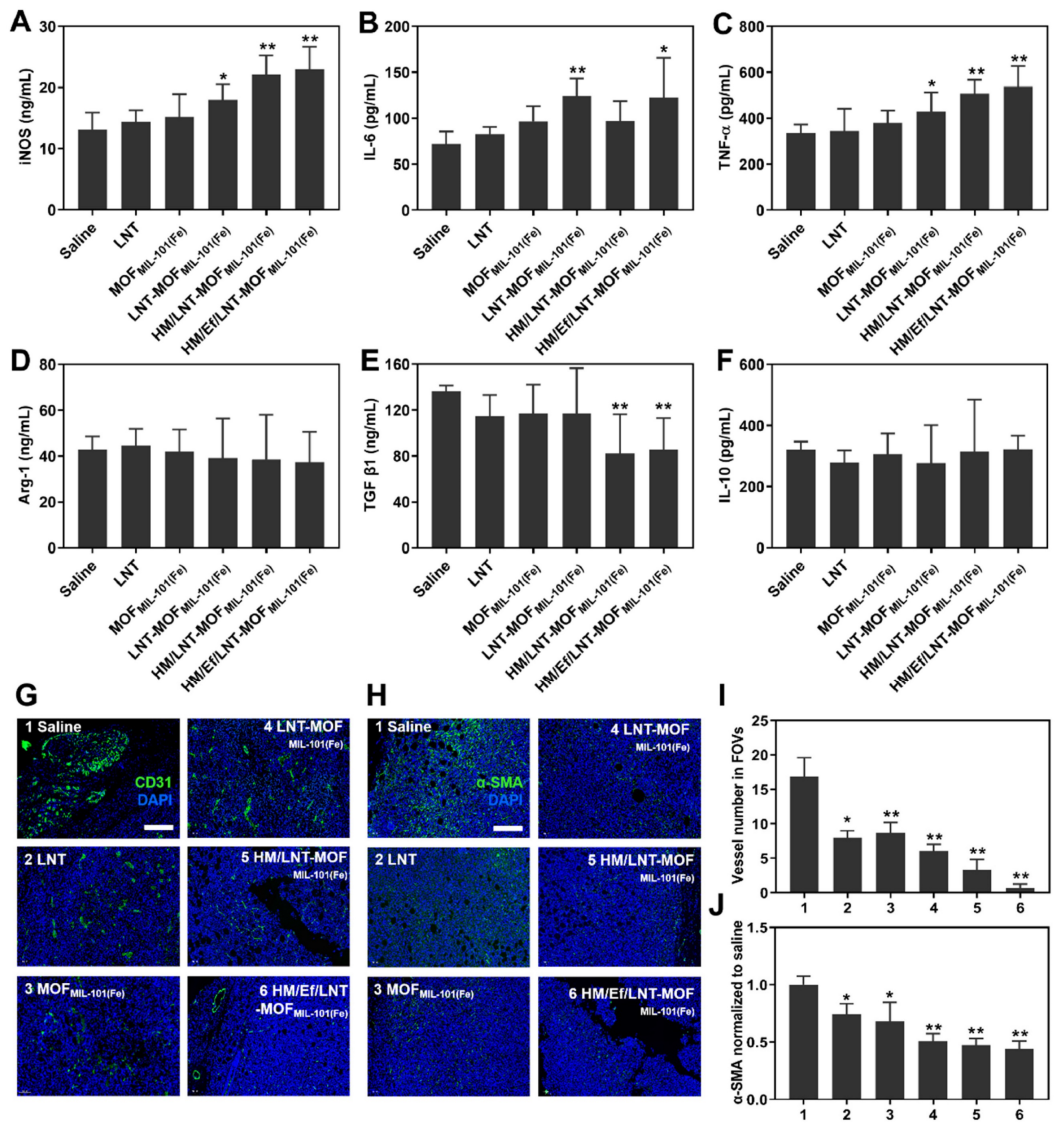
Influence on cytokines and non-immune cells *in vivo*. Serum (A) iNOS, (B) IL-6, (C) TNF-α, (D) Arg-1, (E) TGF-β1, and (F) IL-6 in mice treated with different formulations. (G) CD31-stained and (H) α-SMA-stained immunofluorescence images of tumor sections. The bar is 100 μm. (I) Vessel number and (J) relative α-SMA expression in four randomly-selective FOVs. The data are shown as mean ± SD, n = 4, *p < 0.05, **p < 0.01 vs. Saline.

Cytokines serve as the ultimate regulators in the TME, acting as both instigators and outcomes of tumorigenesis. Therefore, evaluating cytokines is crucial for understanding the TME. In Figure 7A, the serum level of iNOS, a typical cytokine secreted by M1-type TAMs, significantly increased in HM/Ef/LNT-MOF_MIL-101(Fe)_ compared to the saline group. Similarly, Th1-type cytokines IL-6 and TNF-α showed marked elevation after HM/Ef/LNT-MOF_MIL-101(Fe)_ treatment compared to the control (Figure 7B and 7C). In contrast, the Th2-type cytokine TGF-β1 was notably downregulated following HM/Ef/LNT-MOF_MIL-101(Fe)_ treatment (Figure 7D ~ 7F). These findings suggest that HM/Ef/LNT-MOF_MIL-101(Fe)_ might induce pro-inflammatory responses in the TME, consistent with its induction of TAMs M1 polarization.

As illustrated in Figure 7G and 7I, HM/Ef/LNT-MOF_MIL-101(Fe)_ effectively reduced the number of blood vessels in tumor tissue, leading to the normalization of vessel morphology.

Previous studies have suggested that increased infiltration of CD4^+^ T cells could facilitate vascular normalization 39. HM/Ef/LNT-MOF_MIL-101(Fe)_ improved the infiltration of helper T cells, potentially by enhancing LNT delivery, thereby normalizing tumor vascular morphology and density. Additionally, the co-delivery of LNT and iron ions significantly downregulated α-SMA expression in tumors (Figure 7H and 7J), indicating a reduction in the proportion of tumor-associated fibroblasts, another factor in TME activation. These results further strengthen the evidence that HM/Ef/LNT-MOF_MIL-101(Fe)_ can effectively activate the TME.

### Combinational therapies

**Figure 8:**
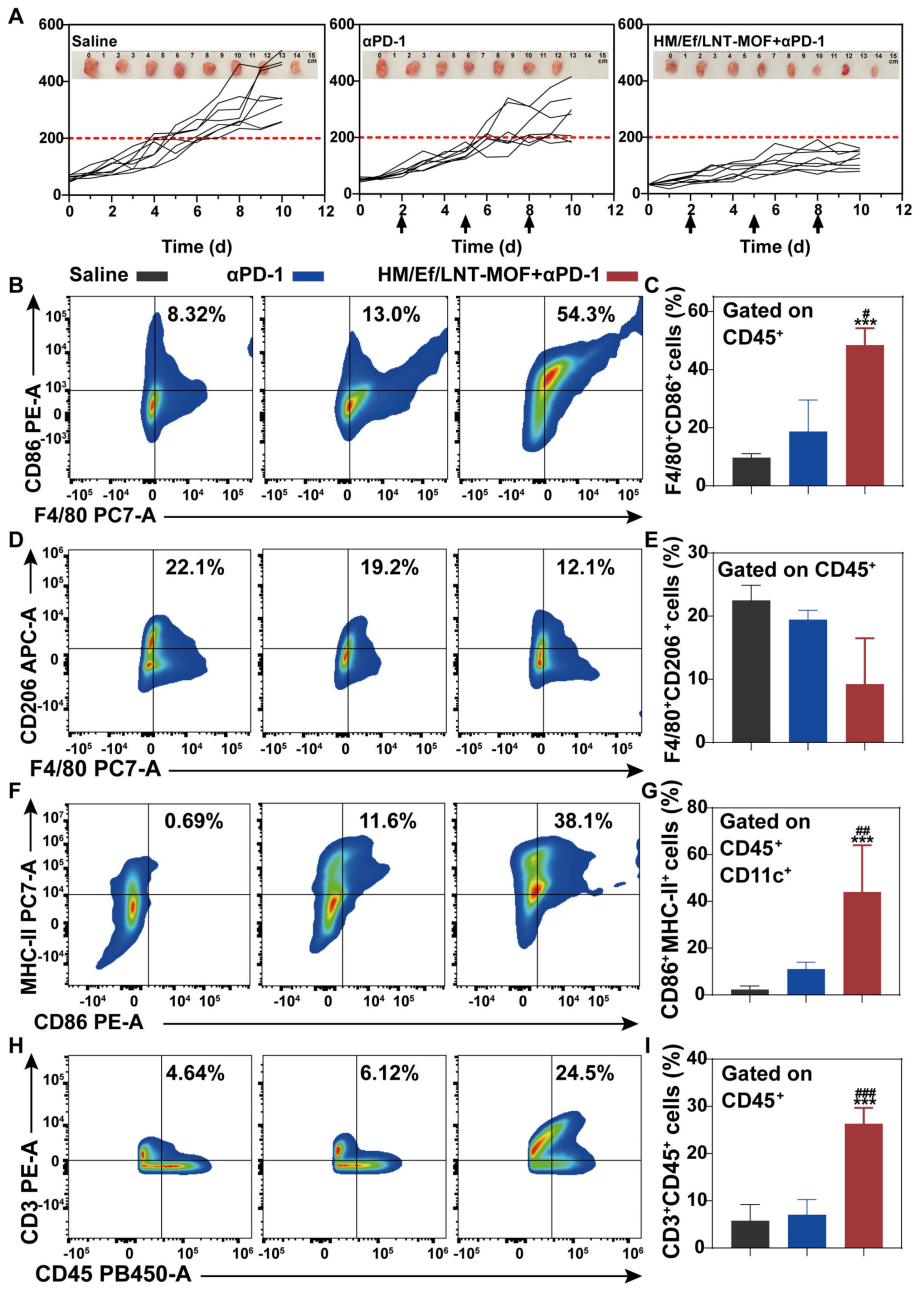
Combinational treatment and TME characterizations. (A) Tumor growth curve of each mouse during the treatments. The arrows present administration time points. The data are shown as mean ± SD, n = 8. The inserted images are *ex vivo* tumors at the end of the treatments. Flow cytometer analysis of (B ~ C) F4/80^+^CD86^+^, (D ~ E) F4/80^+^CD206^+^, (F ~ G) CD86^+^CD11b^+^MHC-II^+^, and (H ~ I) CD3^+^ expression in TME gated on CD45^+^ cell populations. The data are shown as mean ± SD, n = 4, **p < 0.01, ***p < 0.001 vs. Saline; ^#^*p* < 0.05; ^##^*p* < 0.01; ^###^*p* < 0.001 vs. αPD-1.

As illustrated in Figure 8A, the overall anticancer efficacy of αPD-1 treatment was unsatisfactory, with mean tumor size exceeding 330 mm^3^, showing no significant difference compared to the saline group. In contrast, after TME activation with HM/Ef/LNT-MOF_MIL-101(Fe)_, the tumor volumes of mice in the HM/Ef/LNT-MOF_MIL-101(Fe)_ + αPD-1 group were suppressed to below 200 mm^3^, demonstrating significantly superior anticancer ability compared to the αPD-1 group. The tumor sections with HE, TUNEL, and Ki-67-immunochemical staining suggested that the combinational treatment remarkably induced tumor necrosis, promoted apoptosis, and inhibited proliferation compared to the αPD-1 treatment (Figure S7).

**Figure 9:**
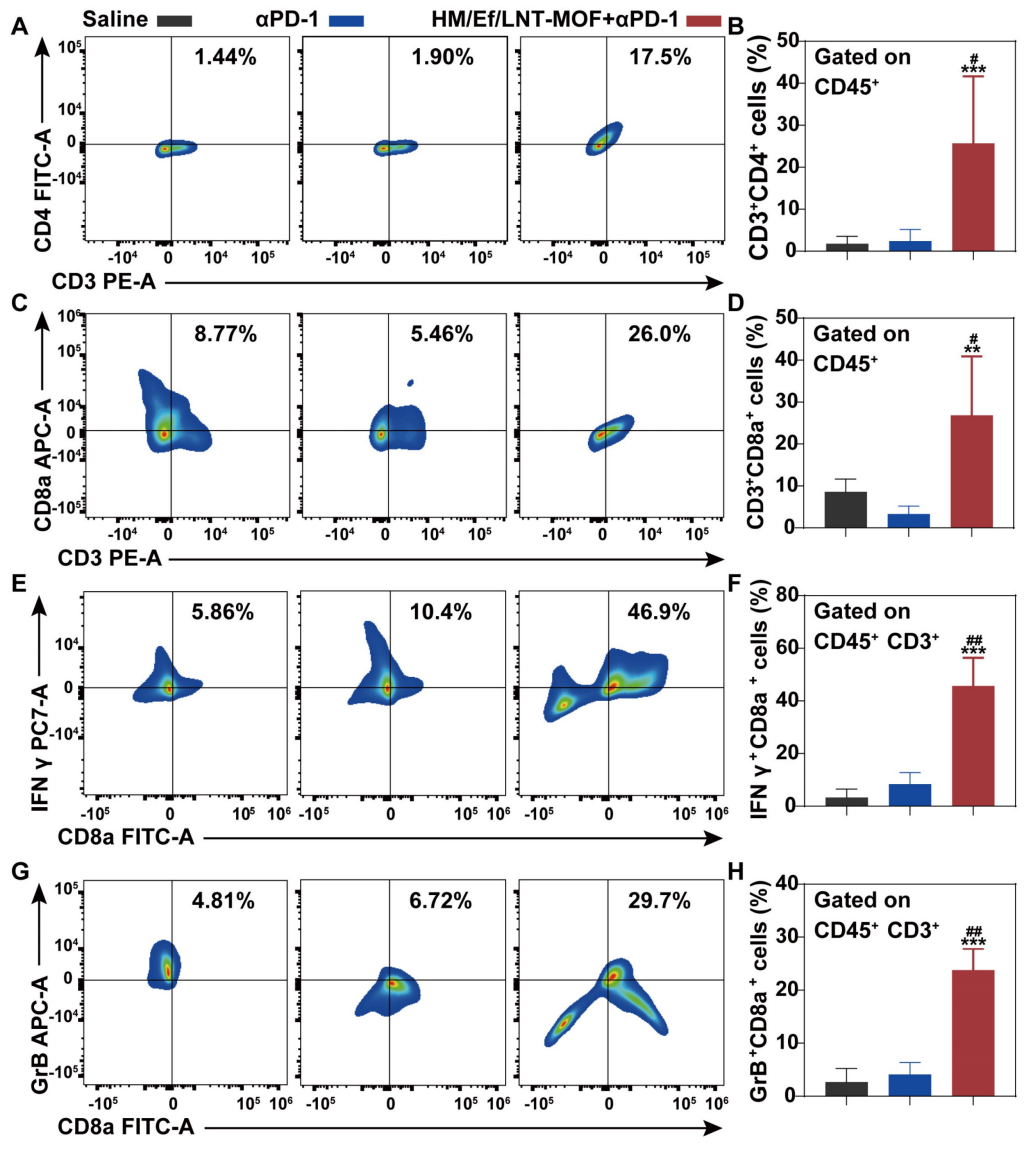
Combinational treatment and TME characterizations. Flow cytometer analysis of (A ~ B) CD3^+^CD4^+^, (C ~ D) CD3^+^CD8a^+^, (E ~ F) CD3^+^CD8a^+^IFN-γ^+^, and (G ~ H) CD3^+^CD8a^+^GrB^+^ expression in TME gated on CD45^+^ cell populations. The data are shown as mean ± SD, n = 4, **p < 0.01, ***p < 0.001 vs. Saline; ^#^*p* < 0.05; ^##^*p* < 0.01; ^###^*p* < 0.001 vs. αPD-1.

As shown in Figure 8B and 8C, flow cytometry analysis of tumor-derived single-cell suspension revealed that the proportion of CD45^+^F4/80^+^CD86^+^ cell populations in the HM/Ef/LNT-MOF_MIL-101(Fe)_ + αPD-1 group achieved 54.3%, which is significantly higher than in the αPD-1 group. Likewise, CD45^+^F4/80^+^CD206^+^ cell populations were also decreased after the combination treatment (Figure 8D and 8E). The combination treatment remarkably elevated the proportion of CD45^+^CD86^+^CD11b^+^MHC-II^+^ cell and total T cell populations compared to the mono αPD-1 group (Figure 8F ~ 8I). As shown in Figure 9A ~ 9D, notable increases in CD4^+^ T cells and CD8a^+^ T cells were observed in the combined group. Moreover, the expression of IFN-γ and granzyme B in CD8^+^ T cells in the HM/Ef/LNT-MOF_MIL-101(Fe)_ + αPD-1 were remarkably higher than αPD-1 alone (Figure 9E and 9F). Such a success of combinational therapy validates the importance of TME activation, with TAMs M1 polarization as the central driving force.

As reported previously, the major issues with anti-TNBC treatment using celastrol-loaded microemulsion (CM) include drug withdrawal rebound and dose-associated toxicity 40. When the CM was combined with HM/Ef/LNT-MOF_MIL-101(Fe)_, as demonstrated in Figure S8, even low-dose and low-frequency CM treatments exhibited excellent tumor suppression, with tumor volumes all below 200 mm^3^, showing a statistical difference compared to CM treatment alone. Furthermore, CM treatment might lead to TAMs polarization toward M2, posing a risk of further immunosuppression in the TME. When HM/Ef/LNT-MOF_MIL-101(Fe)_ was combined with CM, the expression of CD86 in TAMs was significantly enhanced, showing a clear trend of M1 polarization. Likewise, the proportion of various subtypes of T cells was also markedly increased.

In conclusion, these results suggest that the TME activation centered on TAMs M1 polarization induced by HM/Ef/LNT-MOF_MIL-101(Fe)_ can reinvigorate previously ineffective treatments for TNBC.

### Safety evaluations

As shown in Figure S9A, the HE staining images of heart, liver, spleen, lung, and kidney sections from Luc-4T1 tumor-bearing mice treated with various formulations revealed no significant lesions compared to the saline group, indicating the good safety of the MOF_MIL-101(Fe)_-associated systems on major normal organs. As demonstrated in Figure S9B and S9C, liver function indicators, such as aspartate aminotransferase (AST) and alanine aminotransferase (ALT), did not display abnormal elevations in tumor-bearing mice across all treatment groups. Renal function indicators, including blood urea nitrogen (BUN) and uric acid (UA), also remained within the normal range in each group (Figure S9D and S9E). Furthermore, the body weight of mice in each group remained stable throughout the treatment period, with no reductions exceeding 15% of the initial range (Figure S9F), reinforcing the acceptable systemic safety. As shown in Figures S9G to S9L, major blood routine parameters in mice exhibited no apparent abnormalities following various treatments. Moreover, compared to the saline group, no significant differences were observed in the liver index (Figure S9M), spleen index (Figure S9N), and kidney index (Figure S9O) among the treatment groups. These comprehensive tests underscore the potentially good safety of HM/Ef/LNT-MOF_MIL-101(Fe)_ within the employed dosage range.

## Conclusion

HM/Ef/LNT-MOF_MIL-101(Fe)_ is fabricated readily with a well-defined physicochemical structure and desired pharmaceutical characteristics. Such a system remains stable during blood circulation, but rapidly triggers airbag burst under the intra-tumoral environment, releasing a sufficient supply of iron ions and LNT at the tumor site. The synergy between these two components establishes a TME activation pathway loop focused on TAMs M1 polarization, offering a broader scope and potential for various combinational therapies for the TNBC.

## Experimental Section

### Preparation of HM/Ef/LNT-MOF_MIL-101(Fe)_

224.6 mg of BDC-NH_2_ (1.24 mmol) and 675.0 mg of FeCl_3_·6H_2_O (2.5 mmol) were dissolved in 15 mL of *N*, *N*-dimethylformamide (DMF). Following a rigorous mechanical stirring at room temperature for 2 h, the mixture was transferred to an autoclave for a 16-h of reaction at 110 °C. After cooling, the resulting crude product was washed thrice with DMF and anhydrous methanol, successively. 764.5 mg of brown powder was obtained after vacuum drying overnight at 40 °C (named MOF_MIL-101(Fe)_, yield: 85.0%) 30. Subsequently, 20 mg of MOF_MIL-101(Fe)_ and 2 mg of LNT were ultrasonically incubated in 10 mL of deionized water at 37 °C for 2 h. After centrifugation at 5000 *g* for 10 min, the precipitate was freeze-dried, gaining LNT-MOF_MIL-101(Fe)_ at 19.6 mg (yield: 89.1%). Finally, 10 mg of LNT-MOF_MIL-101(Fe)_, 5 mg of NaHCO_3_, and 10 mg of red blood cell&4T1 cell hybrid membrane were sonicated in 10 mL of ultra-purified water for 1 h. Following centrifugation at 3000* g* for 5 min, the precipitate was vacuum-dried to obtain light brown HM/Ef/LNT-MOF_MIL-101(Fe)_ powder at 18.2 mg (yield: 72.8%).

To quantify the content of iron, LNT, and hybrid membrane, HM/Ef/LNT-MOF_MIL-101(Fe)_ was sonicated in pH 4.5 for 2 h and carried out using iron assay kits (Solarbio, BC1735), anthrone-sulfuric acid method 41, and BCA protein assay kit, respectively. The HM composition was assayed by sodium dodecyl sulfate-polyacrylamide gel electrophoresis (SDS-PAGE).

For localization of LNT, the hybrid membrane, and MOF of HM/Ef/LNT-MOF_MIL-101(Fe)_, 0.5 wt% FITC pre-labeled LNT, 0.2 wt% DiI pre-incubated hybrid membrane, and 0.5 wt% Cy5.5-NHS ester pre-incubated MOF_MIL-101(Fe)_ were used to prepare various probe-labeled particles.

### Physicochemical characterizations

The average particle size and zeta potential of diverse MOF_MIL-101(Fe)_ were determined using a dynamic light scattering (DLS) analyzer (NanoZS 90, Malvern, UK). Transmission electron microscopy (TEM, JEOLJEM-1011, Japan) and scanning electron microscopy (SEM, JCM6000, Japan) were employed for morphological investigations. Additionally, 15 mg of various samples underwent analysis using an XD-3A powder diffraction meter with Cu Kα radiation at 40 kV voltage and 30 mA current. Nitrogen adsorption-desorption behaviors of the MOF_MIL-101(Fe)_ were assessed with a specific surface area tester (F-Sorb 2400-BET, China). 1 mL of HM/Ef/LNT-MOF_MIL-101(Fe)_ and MOF_MIL-101(Fe)_ were added to the well of the agarose gel. Upon exposure to different pH stimuli, videos were captured in 2D and contrast imaging modes over a period of 90 s using a Simons diagnostic ultrasound instrument equipped with a high-frequency probe (ML4-20, frequency 14 MHz).

### Fluorescence resonance energy transfer (FRET) analysis

To investigate the spatial relationship between LNT and the hybrid membrane within HM/Ef/LNT-MOF_MIL-101(Fe)_, we employed FITC (5.0 μM) and DiI (2.0 μM), a donor-acceptor FRET pair, to label the LNT and hybrid membrane, respectively. The formulation was positioned in a 24-well plate, and the fluorescence was monitored at predetermined intervals using a microplate reader (Varioskan Flash, Thermo). The FRET ratio was calculated as [I_FRET_ / (I_FRET_ + I_FITC_)] 41, where I_FITC_ and I_FRET_ denote the fluorescence intensity of the FITC and FRET channels, respectively.

### Quartz crystal microbalance (QCM) monitoring

The potential disassembly of HM/Ef/LNT-MOF_MIL-101(Fe)_ under pH stimulations was assessed using QCM. Before experiments, the sample was thoroughly adsorbed onto gold chips in QCM-D cells. The QCM (E1) instrument from Q-Sense operated at room temperature, and the third overtone was utilized to record frequency change (*ΔF*) 42. Once a stable frequency and dissipation were achieved, PBS with varying pH values was successively injected into the QCM-D cells for 30 min to observe changes in frequency.

### Release profile* in vitro*

One microliter of each formulation with equivalent concentrations was placed in a dialysis bag (10 kDa cut-off) and immersed in 100 mL of phosphate buffer (PB) with varied pH values. At predetermined intervals, 1 mL of each sample was withdrawn, filtered through a polycarbonate membrane, and quantified using iron detection kits (Solarbio, BC1735). Subsequently, after collecting and centrifuging the sample within the dialysis bag, the LNT content in the supernatant was quantified using the anthrone sulfate method.

### Intracellular delivery

RAW264.7 (5 × 10^5^) or 4T1 (1 × 10^5^) cells were seeded on polylysine-coated glass sheets (Invitrogen, USA) in 12-well plates and treated with various formulations for 4 h. FITC (5.0 μM) and DiD (2.0 μM) label the LNT and hybrid membrane, respectively. Intracellular endo/lysosomes were stained with 150 nM of LysoTrackerTM Red (Beyotime, C1046, China) for 30 min at room temperature 43. Finally, the cells were stained with DAPI. After rinsing, fixing with 4% paraformaldehyde, the cells were observed immediately by confocal laser scanning microscopy (CLSM, TCS SP8, Leica, Germany).

### Macrophage polarization *in vitro*

RAW264.7 cells (1 × 10^6^ per well in 12-well plates) were treated with different formulations. LPS + IFN-γ (10 pg/mL + 20 ng/mL) and IL-4 (20 ng/mL) treated cells served as M1 and M2 macrophage models, respectively 44. Subsequently, the cells were treated with different formulations for 24 h (the concentration of LNT and iron was normalized to 100 μg/mL and 160 μg/mL, respectively). After 24 h, the cells were stained with anti-F4/80-PE antibody (BioLegend, 111604) and anti-CD86-APC antibody (BioLegend, 105012) in the dark at 4 °C, followed by assaying with a flow cytometry (CytoFLEX, BECKMAN COULTER, UK).

### Cell coculture studies

CTLL-2 cells (1 × 10^5^) were treated with 100 μg/mL LNT for 48 h to obtain conditional medium. Subsequently, 1 × 10^4^ RAW264.7 cells and 2 × 10^4^ 4T1 cells were respectively seeded in the upper and lower chambers with the above conditional medium using a transwell system with a 0.8 μm-sized microporous membrane. RAW264.7 cells were incubated with various formulations at an iron concentration of 160 μg/mL (or LNT concentration of 100 μg/mL) for 24 h. The concentration of MDA and ROS (H_2_O_2_ and OH·) in the culture medium were measured using MDA kit (Beyotime, S0131M), hydrogen peroxide colorimetric detection kit (Enzo Life Science, ADI907015), and 3'-(phydroxyphenyl) fluorescein hydroxyl radical kit (ThermoFisher Scientific, H36004), respectively. CD86 expression of RAW264.7 cells were assayed following the above-mentioned method. Cytotoxicity of 4T1 cells in the lower chamber was assessed using the classic MTT method. Prussian blue staining was performed at room temperature using the classic method on RAW264.7 cells for 24 h to observe morphology and iron uptake.

### Western blot assay

The samples were lysed, centrifuged, and quantified with a BCA Protein Assay Kit. The sample lysate with loading buffer was heated at 100 °C for 10 min and tested with a PowerPacTM HC electrophoresis instrument (BIO-RAD, USA). Subsequently, samples were incubated overnight at 4 °C with primary antibodies against GPX-4 (22 kDa, 1:1000, ab125066), β-tubulin (55 kDa, 1:10 000, Fdbio, FD0064), Caspase-3 (32 kDa, 1:1000, ab184787), GAPDH (36 kDa, 1:10 000, ab181602) and secondary antibodies (1:5000, Immunoway, RS0001) at room temperature for 2 h. The polyvinylidene fluoride membrane, pretreated with an enhanced chemiluminescence kit, was exposed and scanned using the Tanon gel imaging system (Tanon5200, China). The expressions of markers were quantified using ImageJ in triplicate.

### Blood circulation uptake

BALB/c mice were intravenously administered with 100 μL of FITC-labeled HM/Ef/LNT-MOF_MIL-101(Fe)_ (the concentration of FITC, LNT and iron was normalized to 5 μM, 100 μg/mL and 160 μg/mL, respectively). At 2 h post-administration, peripheral blood mononuclear cells (PBMCs) were isolated using Ficoll-PaqueTM PREMIUM. Subsequently, the PBMCs were incubated with anti-mouse CD16/32 antibody (BioLegend, 156604) for 15 min, and then stained with anti-CD11b-PE (BioLegend, 101208) and anti-Ly6G-APC (BioLegend, 127614) antibodies 45. Afterward, flow cytometry (CytoFLEX, BECKMAN COULTER, UK) was immediately employed for analysis.

### Quantification of tumor accumulation

Luc-4T1 tumor-bearing mice were intravenously administered with 100 μL of FITC-labeled HM/Ef/LNT-MOF_MIL-101(Fe)_ (the concentration of FITC, LNT and iron was normalized to 5 μM, 100 μg/mL and 160 μg/mL, respectively) After 24 h, tumors were collected, cut, and digested with 0.1 wt % collagenase type IV, 0.01 wt% hyaluronidase, and 0.02 wt% DNase I 46. The obtained single-cell suspension was filtered and analyzed by flow cytometry to quantify FITC. Iron content in the cells was measured using iron detection kits (Solarbio, BC1735).

### Tumor biodistribution

Luc-4T1 tumor-bearing mice were intravenously administered with 100 μL of FITC/Cy5.5-dual labeled HM/Ef/LNT-MOF_MIL-101(Fe)_ containing 5 μM FITC and 8 μM Cy5.5. After 24 h, tumors were collected, sliced, and analyzed by confocal laser scanning microscopy (CLSM, TCS SP8, Leica, Germany).

### Near-infrared (NIR) imaging *in vivo*

Ten minutes before imaging, Luc-4T1-bearing mice were intraperitoneally administered with 2 mg of *D*-luciferin sodium under online isoflurane anesthesia 47. Bioluminescence was acquired using the IVIS/LuminaXR imaging system (PerkinElmer, USA). Region-of-interest (ROI) tool was used to quantify the bioluminescence of the tumor sites.

### Antitumor efficacy *in vivo*

Luc-4T1-bearing mice with tumor volumes around 40 ~ 60 mm^3^ were randomly divided and intravenously injected with various formulations. The dose of MOF_MIL-101(Fe)_ and LNT was set at 9 mg/kg and 1 mg/kg, respectively. For combination therapies, the dose of celastrol and αPD-1 was set at 2.5 mg/kg 48 and 5 mg/kg 49, respectively. Formulations were administered daily, except for CM and αPD-1, which were given every three days. Body weight and tumor size were recorded once every day. At day 11 post-treatment, blood samples were collected for blood routine (whole blood), biochemical tests (serum), and ELISA assays (serum). Tumor inhibition rate (TIR) was calculated using the formula: TIR (%) = (1 - Tumor_control_/Tumor_treatment_) × 100.

### Pathological section assay

At day 11 post-treatment, mice were sacrificed, and tumors and normal tissues were harvested, weighed, and fixed with formalin. Subsequently, tumor sections were subjected to HE staining, TUNEL-immunohistochemistry, and Ki-67-immunohistochemistry, respectively, according to classic procedure. Normal tissue HE staining followed a standard protocol. Imaging was performed using a fluorescence inverted microscope (VHY-700, Olympus, Japan).

### Flow cytometer studies

A single-cell suspension was prepared from *ex vivo* tumors, following established protocols 46. The suspension was filtered using a 40 µm cell strainer and rinsed with 2 v% FBS. Erythrocytes were lysed with a red blood cell lysis buffer for 5 min. After thorough washing, 100 µL of the cell suspension containing 2 × 10^8^ cells was initially stained with a Zombie NIRTM fixable viability dye (BioLegend, 423106) for 15 min, and subsequently incubated with an FC receptor blocker (anti-mouse CD16/32 antibody, TruStain FcX™, 156604) for an additional 10 min. Afterward, the cells were incubated with various antibodies, including APC anti-human/mouse Granzyme B Recombinant antibody (BioLegend, 372204), PE/Cyanine7 anti-mouse IFN-γ antibody (BioLegend, 505826), FITC Rat Anti-Mouse CD8a (BD Biosciences, 553030), APC anti-mouse CD206 (MMR) antibody (BioLegend, 141708), PE/Cyanine7 anti-mouse I-A/I-E antibody (BioLegend, 107629), Zombie Aqua™ Fixable Viability Kit (BioLegend, 423102), BV421 Rat anti-mouse CD45 (BD Biosciences, 563890), PE anti-mouse CD3 antibody (BioLegend, 100206), PE/Cyanine7 anti-mouse F4/80 antibody (BioLegend, 123114), PE anti-CD86 antibody (BioLegend, 200307), FITC anti-mouse CD4 antibody (BioLegend, 100510), APC anti-mouse CD8a antibody (BioLegend, 100712), and APC anti-mouse CD11c antibody (BioLegend, 117310) at 4 °C in the dark. Moreover, 100 µL of the cell suspension containing 2 × 10^8^ cells was initially stained with DCFH-DA (3 μM, Beyotime, S0033S-1) in the dark. After staining for 30 min, the cells were washed twice with PBS and immediately assayed by flow cytometry (CytoFLEX, BECKMAN COULTER, UK).

### Data analysis

Data were presented as mean ± SD and analyzed for statistical significance using the double-tail Student's *t*-test (comparison between two groups) and ANOVA (comparison among multigroup). Statistical significance was set at ^***^p < 0.001, ^**^p < 0.01, and ^*^p < 0.05.

## Supplementary Material

Supplementary materials and methods, figures.

## Abbreviations

ALT: alanine aminotransferase
Arg-1: arginase-1
AST: aspartate aminotransferase
BDC-NH_2_: 2-aminoterephthalic acid
BUN: blood urea nitrogen
CCR: (C-C motif) receptor
CLSM: confocal laser scanning microscopy
CM: celastrol-loaded microemulsion
DCs: dendritic cells
DMEM: dulbecco's modified eagle medium
DMF: *N*, *N*-dimethylformamide
DLS: dynamic light scattering
Ef: effervescent component -sodium bicarbonate
ELISA: enzyme-linked immunosorbent assay
FTIC: fluorescein isothiocyanate
FRET: fluorescence resonance energy transfer
GSH: glutathione
GPX-4: glutathione peroxidase-4
HM: 4T1&red blood cell hybrid membrane
iNOS: inducible nitric oxide synthase
IFN-γ: interferon-γ
L-6: interleukin-6
IL-10: interleukin-10
LNT: lentinan
LPO: lipid peroxides
LPS: lipopolysaccharide
MOF_MIL-101(Fe)_: MIL-101 metal-organic frameworks
NIR: Near-infrared
PB: phosphate buffer
PBMCs: peripheral blood mononuclear cells
QCM: quartz crystal microbalance
RES: reticuloendothelial system
ROI: region-of-interest
SEM: scanning electron microscopy
SDS-PAGE: sodium dodecyl sulfate-polyacrylamide gel electrophoresis
TAAs: tumor-associated antigens
TAMs: tumor associated macrophages
TME: tumor microenvironment
TNBC: triple negative breast cancer
TME: tumor microenvironment
T_reg_ cells: regulatory T cells
TNF-α: tumor necrosis factor-alpha
TGF-β1: transforming growth factor-beta 1
TEM: transmission electron microscopy
UA: uric acid
