# Supplementary material for: Air bag-embedded MIL-101(Fe) metal-organic frameworks for an amplified tumor microenvironment activation loop through strategic delivery of iron ions and lentinan
Source: Theranostics. 2024 Sep 9;14(15):5883–902. doi: 10.7150/thno.99303 (PMC11426244; doi:10.7150/thno.99303)
Supplement: Supplementary file 1 — Supplementary materials and methods, figures. [file thnov14p5883s1.pdf]

Supplemental Information

Air bag-embedded MIL-101(Fe) metal-organic frameworks for an amplified tumor microenvironment activation loop through strategic delivery of iron ions and lentinan

Tao Han[1,2#], Yan Sun[3#], Xi Jiang[1#], Chengming Gong[1], Fei Kong[1], Yi Luo[1,2], Chang Ge[1], Congyan Liu[1,2], Yuping Liu[1,2], Yanfei Mou[1], Huangqin Zhang[1,2], Jianming Ju[1,2*], Yan Chen[1,2*], Ding Qu[1,2*]

[1] Affiliated Hospital of Integrated Traditional Chinese and Western Medicine, Nanjing University of Chinese Medicine, Nanjing, 210028, China.

[2] Jiangsu Province Academy of Traditional Chinese Medicine, Nanjing, 210028, China.

[3] Jiangsu Cancer Hospital, Nanjing, 210002, China.

[*]Corresponding author: Ding Qu, Ph.D., Professor, Jiangsu Province Academy of Chinese Medicine, 100 Shizi Road, Nanjing 210028, China; Yan Chen, Ph.D., Professor, Jiangsu Province Academy of Chinese Medicine, 100 Shizi Road, Nanjing 210028, China; Jianming Ju, Ph.D., Professor, Jiangsu Province Academy of Chinese Medicine, 100 Shizi Road, Nanjing 210028, China.

[*]Correspondence to: Ding Qu (E-mail: quding1985@hotmail.com); Yan Chen (E-mail: ychen202@hotmail.com); Jianming Ju (E-mail: jjm405@sina.com).

[#]These authors contributed equally to this work.

Materials

A commercial LNT injection with a concentration of 0.5 mg/mL was obtained from Jinling Pharmaceutical Co., Ltd (Jiangsu, China). The LNT, with a molecular weight of $4 \sim 8 \times 10^5$ Da, features a backbone structure of β-(1→3) (1→6)-D-Glu linkage. Iron (III) chloride hexahydrate ($FeCl_3 \cdot 6H_2O$) and 2-aminoterephthalic acid ($BDC\text{-}NH_2$) were sourced from Aladdin Chemical Reagent Co., Ltd (Shanghai, China). InVivoMab anti-mouse αPD-1 (CD279) was procured from BioXcell Co., Ltd. (NH, USA). Celastrol-loaded microemulsion (CM) was prepared by our group,[39] containing a celastrol concentration of 5 mg/mL. Fluorescein isothiocyanate (FTIC), DiD, and DiI were acquired from SinoPharma Co., Ltd (Shanghai, China). Cyanine5.5 (Cy5.5®) NHS ester was obtained from Lumiprobe Limited (HK, China). The GSH Assay Kit (ab239727), anti-glutathione peroxidase-4 (GPX-4) antibody (ab252833), anti-caspase-3 antibody (ab13847), mouse inducible nitric oxide synthase (iNOS) enzyme-linked immunosorbent assay (ELISA) kit (ab253219), L-Lactate ELISA kit (ab65330), mouse interferon-gamma (IFN-γ) ELISA kit (ab282874), and mouse arginase-1 (Arg-1) ELISA kit (ab269541) were sourced from Abcam (Shanghai, China). Mouse interleukin-6 (IL-6, KGEMC004), tumor necrosis factor-alpha (TNF-α, KGEMC102a), interleukin-10 (IL-10, KGEMC005), and transforming growth factor-beta 1 (TGF-β1, KGEMC107b) ELISA kits were purchased from Nanjing KeyGen Biotech Co., Ltd (Nanjing, China). Lipopolysaccharide (LPS) was provided by Sigma-Aldrich Chemie GmbH (WI, Germany). Murine recombinant IL-4 was offered by PeproTech (NJ, USA). Purified water was generated using the Millipore Elix 5 system (MA, USA).

Animals

Female BALB/c mice (6 ~ 8 weeks, 22 ~ 24 g) were sourced from Changzhou Cavens Laboratory Animal Co. Ltd. (Jiangsu, China). These mice were maintained in a pathogen-free environment and provided with ad libitum access to food and water under specific pathogen-free (SPF) conditions. The anesthesia process was performed by the online isoflurane anesthesia method with channel-3 flow. All experimental procedures involving animals strictly adhered to the Guidelines for Care and Use of Laboratory

Animals and received approval from Jiangsu Province Academy of Traditional Chinese Medicine Animal Experimentation Ethics Committee (approval number: AEWC-20181207-67).

Cell culture

4T1 and RAW264.7 cells were cultured in Dulbecco's Modified Eagle Medium (DMEM) with 10% (v/v) FBS and 50 μg/mL penicillin/streptomycin in a humidified incubator (Thermo 3111, Thermo Fisher Scientific, USA) with 5% CO_2 at 37°C. CTLL-2 cells followed a similar protocol but utilized RPMI 1640 medium.

Drug loading (DL) of HM/Ef/LNT-MOF$_{MIL-101(Fe)}$

To quantify the content of iron, LNT, and the hybrid membrane (HM), the HM/Ef/LNT-MOF$_{MIL-101(Fe)}$ was sonicated in a pH 4.5 buffer for 2 h. The iron was measured using iron assay kits (Solarbio, BC1735), the LNT was determined by the anthrone-sulfuric acid method, and the protein content was assessed using the BCA protein assay kit. For the determination of $NaHCO_3$ content, the HM/Ef/LNT-MOFMIL-101(Fe) was dissolved in deionized water and quantified HCO_3^- using acid-base titration.

Cellular uptake

One hundred thousand RAW264.7 or 4T1 cells were seeded per well in 12-well plates and treated with various formulations for 4 h. FITC and iron concentrations were maintained at 5.0 μM and 400 μg/mL, respectively. After rinses thrice, cells were prepared as single-cell suspensions, and fluorescence quantification was performed using a cytometer (CytoFLEX, BECKMAN COULTER, UK). The cells were lysed to measure the iron content with iron detection kits (Solarbio, BC1735).

Biodistribution of iron

The biodistribution of iron in Luc-4T1 tumor-bearing mice was assessed following intravenous administration of 100 μL of different formulations, with the concentrations of LNT and iron normalized to 100 μg/mL and 160 μg/mL, respectively. Normal tissues

(heart, liver, spleen, lung, kidney) and tumors were collected and homogenized at 6 h and 24 h post-administration. The homogenates were diluted with two volumes of saline and centrifuged at 5000 *g* for 10 min. The iron content in the supernatant was measured using iron detection kits (Solarbio, BC1735).

Xenograft tumor models

A suspension of Luc-4T1 cells (2×10^6 cells, 100 μL) was injected into the fourth mammary fat pad of female BALB/c mice following a classic protocol. Tumor size was measured daily from day 3 post-implantation, and tumor volume was calculated using the formula: V (mm^3) = $W^2 \times L/2$, where L and W represent the length and width of tumor, respectively.

Table S1. Pharmaceutical characterizations of various MOF formulations. Data are presented as mean ± SD; n = 4.

Formulations	DL of LNT (%)	DL of $NaHCO_3$ (%)	Iron ions (wt %)	Protein (wt%)
LNT-MOF$_{MIL\text{-}101(Fe)}$	2.7 ± 0.3	NA	4.2 ± 0.3	NA
HM/LNT-MOF$_{MIL\text{-}101(Fe)}$	2.9 ± 0.2	NA	4.1 ± 0.2	22.4 ± 1.3
HM/Ef/LNT-MOF$_{MIL\text{-}101(Fe)}$	3.1 ± 0.3	14.5 ± 2.4	4.6 ± 0.3	23.5 ± 2.1

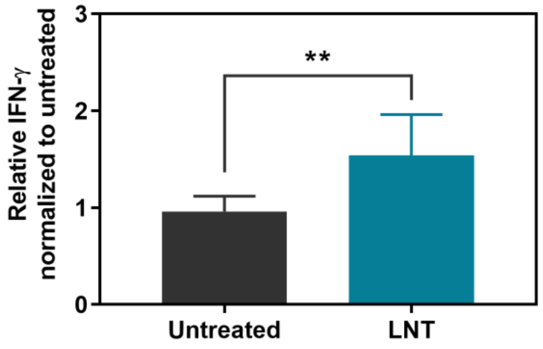

Figure S1. Relative IFN-γ secretion of CTLL-2 cells after treatment with LNT for 48 h. Data are

presented as mean ± SD; n = 3. $^{**}P < 0.01$.

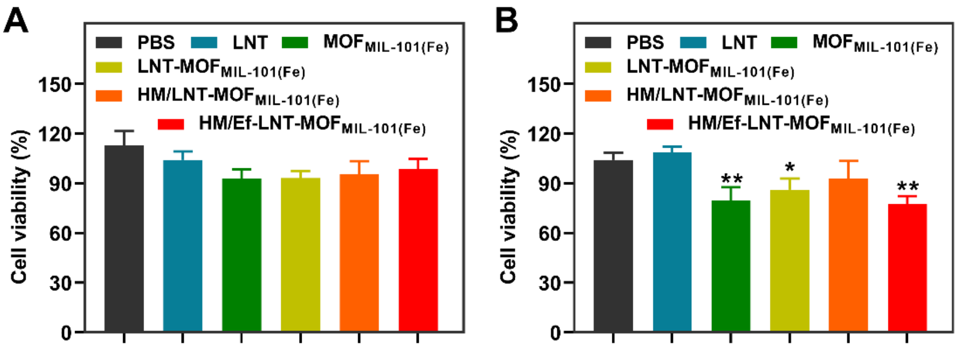

Figure S2. Cytotoxicity of (A) RAW264.7 cells and (B) 4T1 cells after treated with different formulations for 24 h. Data are presented as mean ± SD; n = 6. $^{*}P < 0.05$, $^{**}P < 0.01$ vs. PBS.

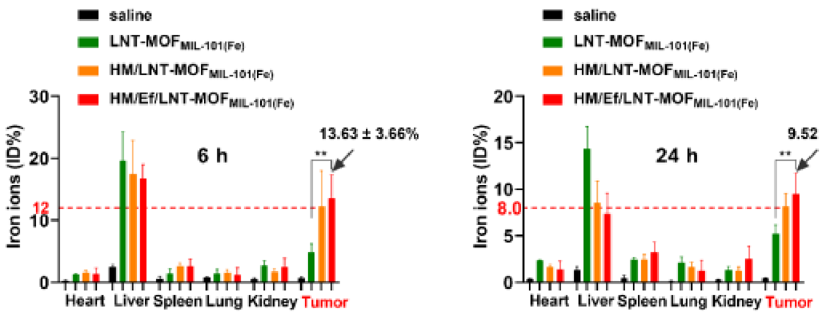

Figure S3. The distribution of iron ions in various tissues, including heart, liver, spleen, lung, and tumor, at 6 h and 24 h post administration. Data are presented as mean ± SD; n = 3. $^{**}P < 0.01$ vs. LNT-$MOF_{MIL\text{-}101(Fe)}$.

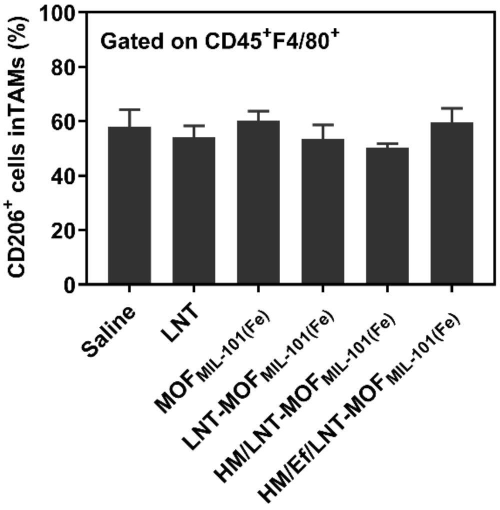

Figure S4. TAMs polarization *in vivo*. Quantification of CD206 expression in TAMs gated on $CD45^+F4/80^+$ cell populations from flow cytometer analysis post-treated with different formulations. Data are presented as mean ± SD; n = 4.

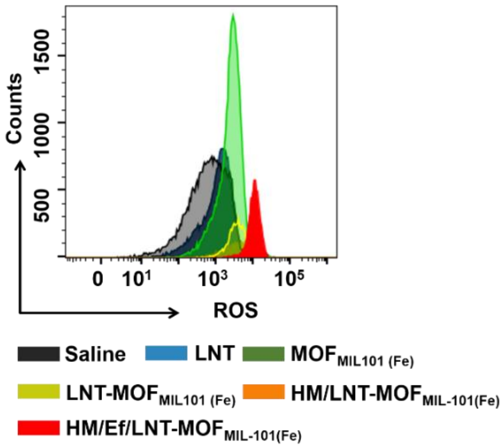

Figure S5. Fluorescence of ROS from tumor-derived single cell suspension, after being treated with different formulations, studied by flow cytometer.

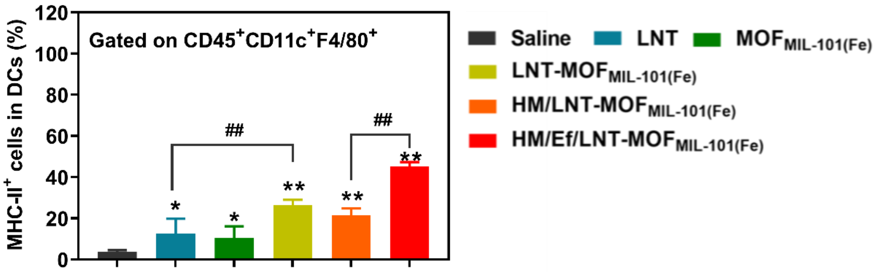

Figure S6. Quantification of MHC-II expression in TAMs gated on $CD45^+F4/80^+CD11c^+$ cell populations from flow cytometer analysis. The data are shown as mean ± SD, n = 4, $^{*}P < 0.05$, $^{**}P < 0.01$ vs. Saline; $^{\#\#}P < 0.01$.

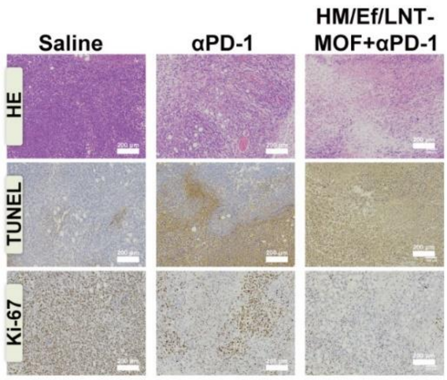

Figure S7. HE staining, TUNEL immunofluorescence, and Ki-67 immunohistochemistry images of tumor sections after different treatments. The scale bar is 100 μm.

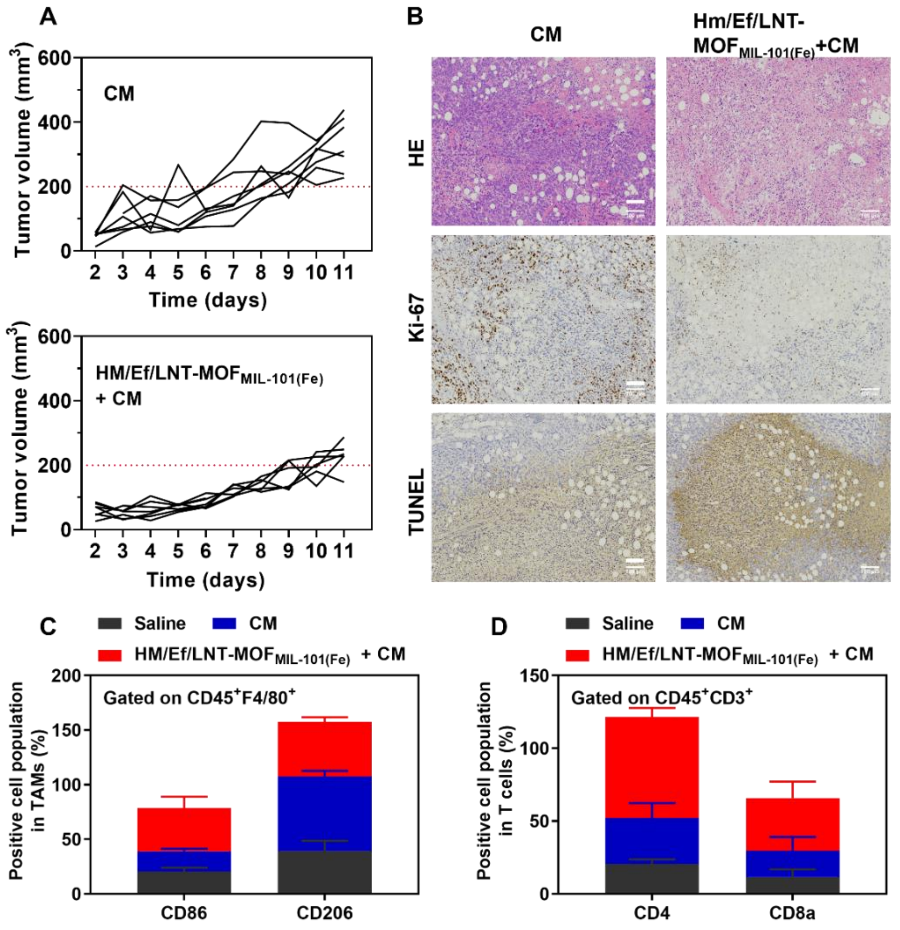

Figure S8. (A) Tumor growth curve of each mouse during the treatments. The data are shown as mean ± SD, n = 6. (B) HE staining, TUNEL immunofluorescence, and Ki67 immunohistochemistry images of tumor sections after the treatments. The scale bar is 100 μm. Flow cytometer analysis of (C) F4/80$^+$CD86$^+$&F4/80$^+$CD206$^+$, and (D) CD3$^+$CD4$^+$&CD3$^+$CD8a$^+$, in TME gated on CD45$^+$ cell populations. The data are shown as mean ± SD, n = 4.

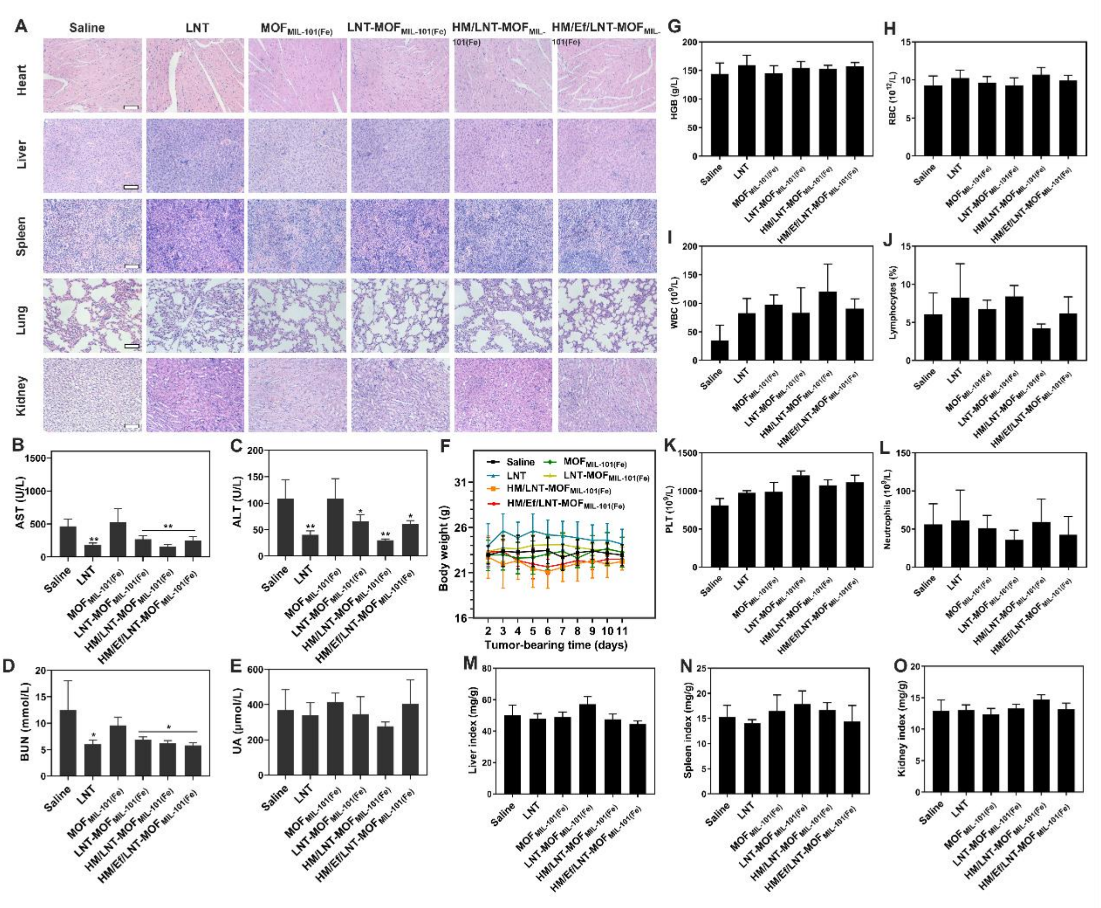

Figure S9. Safety evaluation *in vivo*. (A) HE-stained images of normal tissues from mice treated with different formulations. The scale bar is 100 μm. Serum (B) AST, (C) ALT, (D) BUN, and (E) UA from each mouse. (F) Body weight of each mouse during the treatments. Determination of (G) HGB, (H) RBC, (I) WBC, (J) lymphocytes, (K) PLT, and (L) neutrophils in whole blood from each mouse. (N) Spleen index and (O) kidney index of the mice. The data are shown as mean ± SD, n = 6, $^{*}P < 0.05$, $^{**}P < 0.01$ vs. Saline.
